# Multiple impacts of microplastics can threaten marine habitat-forming species

**DOI:** 10.1038/s42003-021-01961-1

**Published:** 2021-03-30

**Authors:** Cinzia Corinaldesi, Sara Canensi, Antonio Dell’Anno, Michael Tangherlini, Iole Di Capua, Stefano Varrella, Trevor J. Willis, Carlo Cerrano, Roberto Danovaro

**Affiliations:** 1 Department of Materials, Environmental Sciences and Urban Planning, Polytechnic University of Marche, Via Brecce Bianche, Ancona, Italy; 2 Department of Life and Environmental Sciences, Polytechnic University of Marche, Ancona, Italy; 3 Department of Research Infrastructures for Marine Biological Resources, Stazione Zoologica Anton Dohrn, Fano Marine Centre, Fano, Italy; 4 Stazione Zoologica Anton Dohrn, Villa Comunale, Naples, Italy; 5 Department of Integrative Marine Ecology, Stazione Zoologica Anton Dohrn, Fano Marine Centre, Fano, Italy

**Keywords:** Ecology, Zoology

## Abstract

Microplastics are recognised as a potential global threat to marine ecosystems, but the biological mechanisms determining their impact on marine life are still largely unknown. Here, we investigated the effects of microplastics on the red coral, a long-lived habitat-forming organism belonging to the *Corallium* genus, which is present at almost all latitudes from shallow-water to deep-sea habitats. When exposed to microplastics, corals preferentially ingest polypropylene, with multiple biological effects, from feeding impairment to mucus production and altered gene expression. Microplastics can alter the coral microbiome directly and indirectly by causing tissue abrasions that allow the proliferation of opportunistic bacteria. These multiple effects suggest that microplastics at the concentrations present in some marine areas and predicted for most oceans in the coming decades, can ultimately cause coral death. Other habitat-forming suspension-feeding species are likely subjected to similar impacts, which may act synergistically with climate-driven events primarily responsible for mass mortalities.

## Abstract (web summary)

Corinaldesi et al. investigated the biological responses to microplastic contamination of the red coral Corallium rubrum, a genus with near global distribution. Microplastics caused feeding impairment, tissue damage, altered gene expression, oxidative DNA damage and microbiome alteration. Such diverse deleterious effects may be generalizable to other habitat-forming suspension and filter feeders given the burgeoning levels of microplastic contamination across the world’s oceans.

## Introduction

Microplastics (i.e., particles <1 mm)^1,2^ present in the marine environment may fall into the optimal range of prey size for a wide range of marine organisms^3^ and can jeopardize different levels of biological organization^4^, thus representing a potential threat to the conservation of biodiversity and ecosystem function.

Microplastic concentrations can vary widely in marine ecosystems depending on environmental and biological factors, and their measurement is influenced by the methodology used to collect and analyse the polymers and their target size^5,6^. Microplastic particles can be found at concentration of thousands of particles per m^3^ in some coastal marine areas^5,7,8^ and are predicted to double, despite recent bans, by 2030^9^. Current estimates indicate that the concentrations of microplastics, which generally are not accurately quantified, due to the need of specific sampling devices and ad hoc methodological protocols, can be several orders of magnitude higher than those reported so far in coastal and offshore ecosystems^10^.

Microplastics can have negative effects on phytoplankton, zooplankton, fish and large marine organisms^11–14^, either at a molecular (e.g., gene expression and production of reactive oxygen species)^15–21^, cellular (e.g., apoptosis, membrane stability)^3,22^ or population level (e.g., reproduction, development, feeding activity)^15,23–28^.

However, available information is still primarily based on toxicological assays^20^ typically carried out using selected polymer microspheres/beads, marked with contaminants or fluorescent probes^7,29^. Since these tests may not reflect natural conditions^20^, there is a need for the development of experimental studies able to mimic natural conditions, combining laboratory controls with multidisciplinary approaches that can provide a more comprehensive understanding of the molecular, cellular, physiological, behavioural and ecological implications^8,20,30^.

Octocorals are sessile colonial invertebrates that include some of the most important ecosystem engineers in temperate areas and in deep sea, contributing to the high levels of biodiversity and ecosystem functions^31^. The genus *Corallium* is distributed worldwide, at almost all latitudes, spanning Antarctica, the northern Pacific (Hawaii, Japan), the southern Pacific (New Zealand, Tasmania), the equatorial latitudes (Taiwan), the eastern Pacific, the western Atlantic and the Mediterranean Sea^32–34^.

Most of the available information on corals suggests that the microplastic particles ingested alter feeding performance^35–37^ and can increase their susceptibility to disease in scleractinian corals^38^.

Here, for the first time, we used replicated experiments in aquaria to explore the multiple impacts of microplastics on the octocoral *Corallium rubrum* (distributed from the Mediterranean Sea to the Atlantic Ocean)^39^ and to investigate the biological mechanisms underpinning its responses. Coral colonies were exposed to microplastic mixtures, selected to reflect the size, polymeric composition and concentrations present in highly contaminated coastal waters^7,8,10^, or predicted for some oceanic regions in the coming decades^9^.

We investigated the responses of *C. rubrum* in terms of feeding activity and defence mechanisms, the tissue damage due to the physical contact with microplastics, responses at the molecular level (i.e., gene expression and DNA damage), and the influence on the coral-associated microbiome.

This study provides new insights into the multiple biological responses of marine life to microplastic contamination and on factors that can ultimately threaten habitat-forming and suspension-feeding species.

## Results

### Feeding activity and microplastics ingestion by *C. rubrum* and its prey

The study tests the effects of three different levels of microplastic contamination (here defined as low, medium and high concentrations of microplastic particles). The microplastic concentrations actually available to *C. rubrum*, determined from the analysis conducted in the CTRL MPS (aquaria containing 1000 microplastic particles L^−1^ but without coral branches), were, on average, 602–633 microplastic particles L^−1^, within the first 14 days of experiments (see Supplementary Results for full details).

The results obtained from the feeding experiments on *C. rubrum* are reported in Supplementary Table 1 and the outputs of the linear regression model in Supplementary Table 2.

**Fig. 1:**
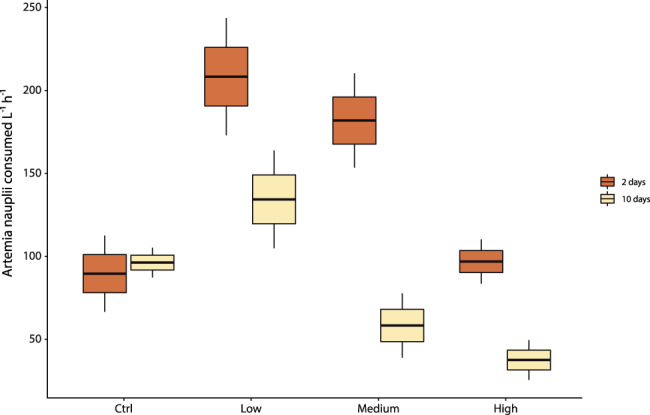
Feeding rates of *C. rubrum* exposed to microplastics. Reported are data obtained by coral branches exposed to different concentrations of microplastics (low, medium and high) and fed with nauplii of *A. salina* after 2 and 10 days of experiment. Data are represented as mean ± standard deviation (*n* = 3).

After 2 days of experiment, the coral polyps exposed to low and medium concentrations of microplastic particles showed a significantly higher ingestion rate of *Artemia salina* compared to the controls (ANOVA F_3,8_ = 15.59, *P* = 0.001, Fig. 1), implying that the increased availability of particles stimulated feeding activity. No significant differences were observed between feeding activity in control and corals exposed to the high concentration of microplastic particles. However, after 10 days, feeding rates in the corals exposed to medium and high concentrations of microplastic particles declined significantly compared to the controls, whereas corals that were exposed to low concentrations of microplastic particles did not feed at significantly different rates from the controls.

Comparing the coral feeding rates after 2 and 10 days of the experiment, we found a significant decline in all treatments, whereas in the controls remained consistent (Fig. 1). The lack of change in the feeding rates in the controls between day 2 and day 10 caused a significant interaction term in a two-way ANOVA testing the effects of Treatment and Time (Treat × Time *F*_3,16_ = 8.1, *P* = 0.0016). To estimate the relative effects of varying plastic concentration, the model was rerun without the controls, resulting in no interaction and significant main effects (Treat × Time *F*_2,12_ = 2.8, *P* = 0.099). The overall effect of Time was a reduction in feeding rate of 59.4 ± 20.1 nauplii L^−1^ h^−1^ between day 2 and day 10 (F_1,12_ = 54.71, *P* < 0.001). Increasing microplastic concentrations also resulted in reductions in feeding rate in tanks containing the highest microplastic concentration, where feeding rate was 37.5 ± 18.9 nauplii L^−1^ h^−1^. The feeding rates at medium and low concentrations of microplastic particles were 20.8 ± 20.0, and 96.9 ± 20.0 nauplii L^−1^ h^−1^, respectively, higher than the treatment with the highest concentration of microplastic particles.

**Fig. 2:**
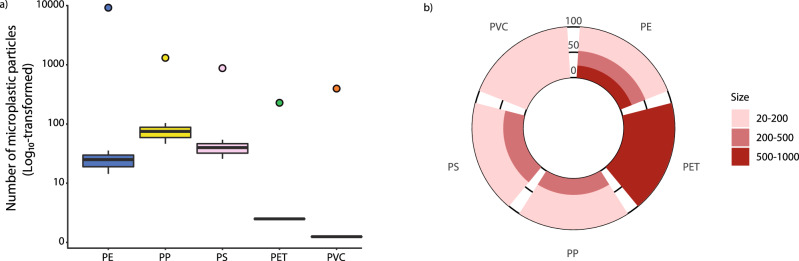
Microplastic particles ingested by the red coral *Corallium rubrum* and their polymer composition. **a** Log_10_-transformed number of different microplastic polymers ingested by *C. rubrum* after 14 days of experiment (average number per branch) in the systems with high concentration of microplastic particles. Circles indicate the Log_10_-transformed number of each polymer added to the tanks at the beginning of the experiment. Data are represented as mean ± standard deviation (*n* = 3). **b** Contribution (expressed as percentage) of different size of microplastics ingested by a branch of *Corallium rubrum*. Abbreviations of plastic polymers: PE polyethylene, PP polypropylene, PS polystyrene, PET polyethylene terephthalate, PVC polyvinylchloride.

After 14 days, corals that were exposed to the highest concentration of microplastic particles, contained on average 143.8 ± 8.8 particles per branch (ca. 0.18 mg, ca. 2.6 particles per polyp, Fig. 2a). The size of microplastics ingested by corals ranged from 20 µm to 1 mm (Fig. 2b) and the type of plastic ingested was not random (F_4,10_ = 12.34, *P* < 0.001). Comparing the same size classes, the most frequently ingested polymers by the red coral were polypropylene (PP), followed by polystyrene (PS) and polyethylene (PE) (Fig. 2a). Polypropylene was the most consumed polymer, although high variation meant that after Bonferroni correction for multiple comparisons only the contrasts with PET and PVC were statistically significant from PP (*P* = 0.001 in both cases).

The *A. salina* nauplii, used as prey to feed *C. rubrum*, also ingested microplastics, which were clearly visible by microscopic analysis in the digestive tract of nauplii (Supplementary Fig. 1a). On average, 0.2 ± 0.01 microplastic particles per individual were ingested by *A. salina* after 2 days of experiment and 1.89 ± 0.44 microplastic particles per individual were ingested by *A. salina* after 10 days of experiment. At the 10th day of the experiment, the size range of microplastics ingested by *A. salina*, recovered after their enzymatic digestion, ranged from 10 to 141 µm, with mean size 43.5 ± 36.7 and modal value 25. Polystyrene was the most abundant plastic polymer ingested by *A. salina* (Supplementary Fig. 1b).

### Physical impact on coral coenenchyma

**Fig. 3:**
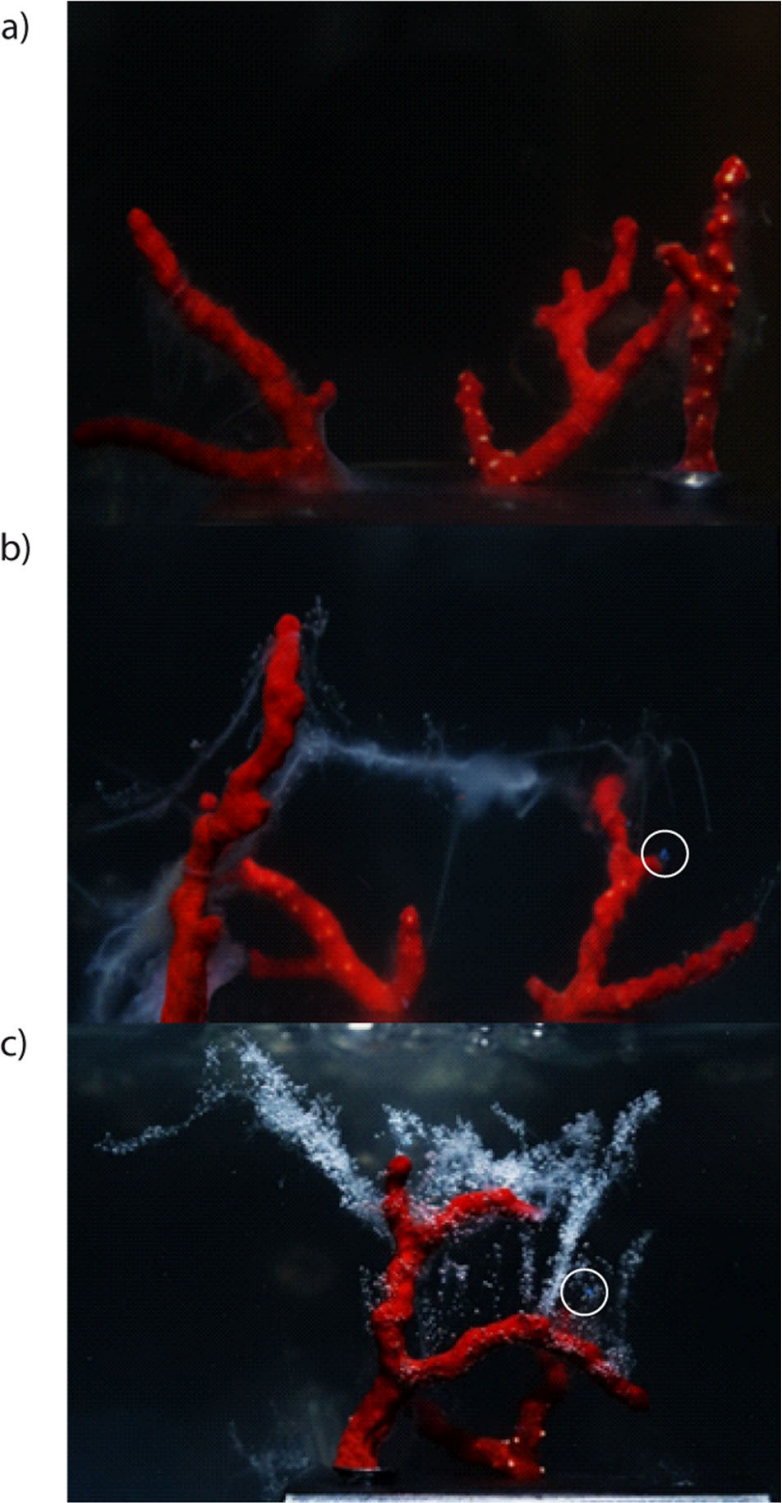
The red coral (*Corallium rubrum*) in the experimental systems exposed to varying concentrations of microplastic particles after 10 days of experiment. **a** Low, **b** medium, and **c** high concentrations of microplastic particles. Mucus release increases with increasing microplastic particles concentration. White circles indicate microplastics items trapped by mucus (polyethylene particles).

The corals exposed to microplastics, especially at the higher concentrations, showed the presence of microplastic particles around coral branches (Fig. 3).

**Fig. 4:**
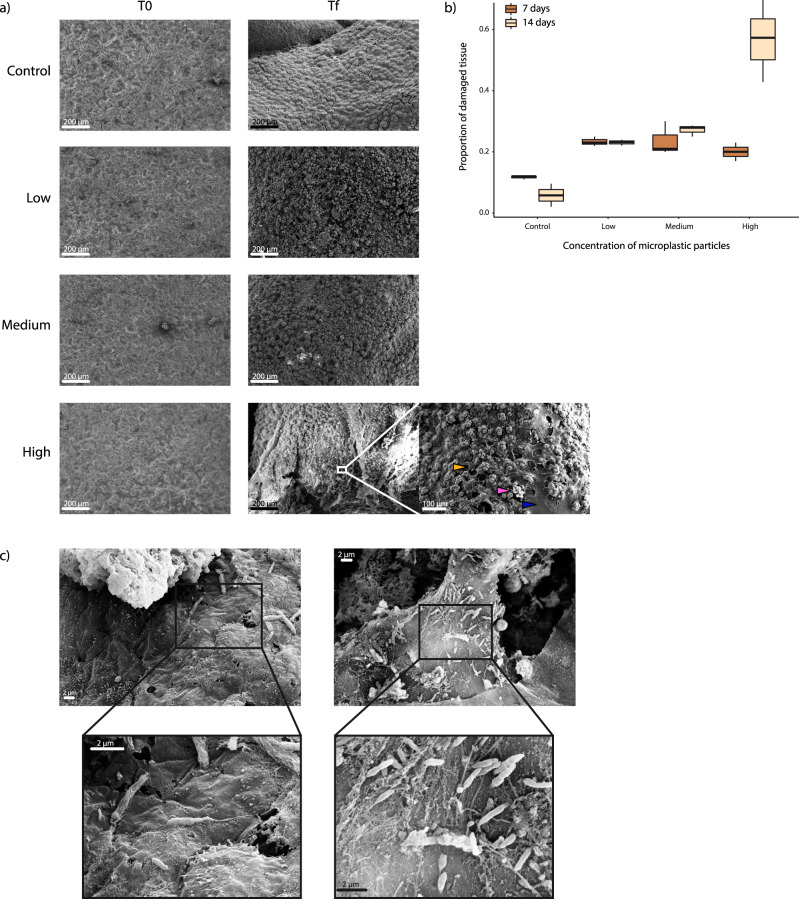
SEM images of tissue damage of *C. rubrum* exposed to different microplastic concentrations and injured coral tissue colonized by bacterial cells. **a** SEM images of the coral tissue exposed and not exposed to microplastics (Control) at the beginning (t0) and at the end of the experiment (tf, i.e., 14 days after the start of the experiment) and a close-up of coral tissues exposed to high concentration of microplastic particles at the end of the experiment (the blue arrow indicates damaged tissue; the yellow arrow indicates healthy tissue and the fuchsia arrow indicates one of the sclerites). Size scales are provided for each picture. **b** Average proportion of damaged tissue area of corals exposed to different microplastic concentrations after 7 and 14 days of treatment. At the start of the experiment (t0), tissue damage was not detectable in all specimens. Data are represented as mean ± standard deviation (*n* = 3). **c** On the left, SEM image of the coenenchyma without lesions in *C. rubrum* exposed to high concentration of microplastic particles and relative close-up of the tissue with evidence of the bacillus-like cells colonizing the tissue; on the right, coral tissue with lesions densely colonized by bacillus-like cells.

Scanning Electron microscopy (SEM) analyses carried out on the coral tissue revealed that, at the beginning of the experiment, no corals showed signs of tissue damage, but after 7 days the extent of damage of the corals exposed to microplastics was approximately double than that of the controls (Fig. 4a, b). At the beginning of the experiment (t0) the portion of damaged tissue both in the control and exposed corals to microplastics was undetectable. After 1 week of experiment the tissue damage significantly increased in all corals, but a damage level ≥20% was evident only in the presence of microplastics. After 14 days, the fraction of damaged tissue was up to 6.5 times higher in the corals exposed to the highest concentrations of microplastics than in the controls (Fig. 4b). Compared to the control, the extent of damage increased with increasing microplastic concentration: 217% when exposed at low concentrations (with lower and upper 95% confidence limits of 207 and 228%), 260% for medium concentrations (248–272%) and 546% for high concentrations (525–568%) of microplastic particles, respectively.

The analysis of prokaryotic abundances in the corals exposed to high concentrations of microplastic particles revealed the presence of significantly higher numbers of prokaryotes close to the lesions than in the intact tissues (two-tailed *t* = 10.88, *P* = 0.004, Fig. 4c, Supplementary Fig. 2).

### Stress signals

#### Mucus release and trapped microplastics and prokaryotic cells

The first negative effects of microplastics at different concentrations on *C. rubrum* were observed a few hours after the beginning of the experiment, as the quantity of mucus produced by the red coral colonies increased with increasing concentrations of microplastics, the mucus aggregated, became denser and persisted for the entire duration of the experiment (Fig. 3).

At the end of the experiment, the corals exposed to the highest concentrations of microplastics, showed ca. 43 particles mL^−1^ (size range 200–1000 µm) within coral mucus released, with an enrichment factor >40 (calculated as the ratio of microplastic abundance in mucus and surrounding seawater present at the beginning of the experiment, vol:vol). The abundances of prokaryotes contained within coral mucus were significantly higher (linear mixed-model *t*_1,18_ = 4.92) at high microplastic concentrations (4.0 ± 0.7 × 10^6^ cells mL^−1^) than at low and medium microplastic concentrations (2.4 ± 0.6 × 10^6^ cells mL^−1^ and 2.3 ± 0.7 × 10^6^ cells mL^−1^, respectively; F_1,16_ = 24.25, *P* < 0.001, Supplementary Fig. 3a).

#### Gene expression and oxidative DNA damage levels

**Fig. 5:**
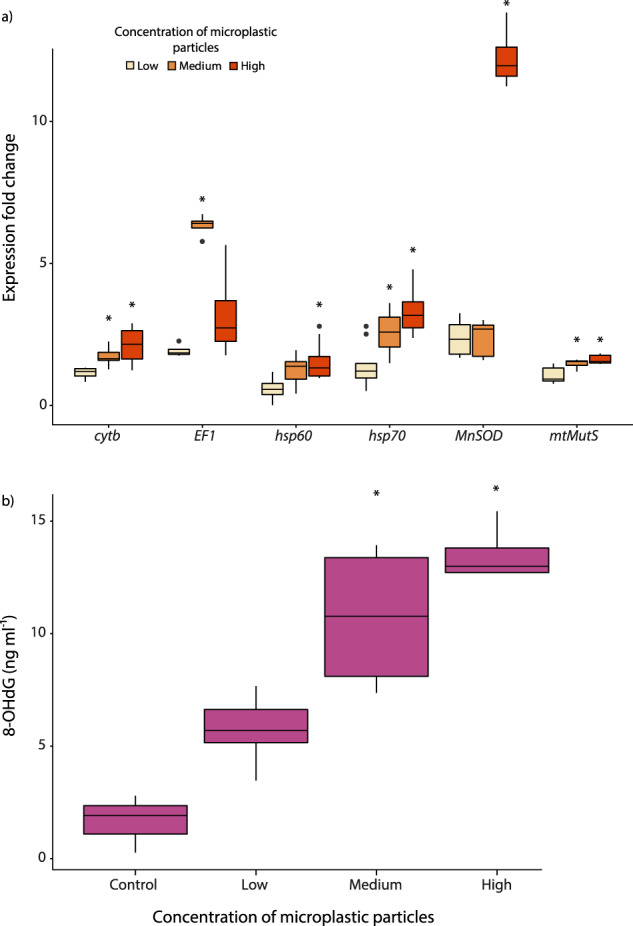
Gene expression patterns and oxidative DNA damage in *C. rubrum* exposed to different levels of microplastic concentrations after 10 days of experimental incubation. **a** Expression fold change of six genes (*cytb, mtMutS, hsp70, hsp60, EF1, MnSOD*) obtained by real-time qPCR; **b** 8-hydroxydeoxyguanosine (*8-OHdG*) levels obtained by ELISA. Average value and standard deviation (±) are reported. Significant differences for the expression of each gene and for 8-OHdG levels compared to the controls (corals not exposed to microplastics) are highlighted with asterisks: *P < 0.05.

The expression levels of *cytb, mtMutS, hsp60, hsp70* and *MnSOD* in the coral branches (*n* = 3) determined immediately before the experiment (t0) did not differ from the expression levels measured in the controls after 10 days of experiment (Supplementary Table 3). In the corals exposed to medium concentration of microplastic particles, the expression levels of *cytb, mtMutS, hsp70* and *EF1* genes were significantly higher than those not exposed to microplastics after 10 days of experiment (statistical details in Supplementary Table 4). At the highest concentration of microplastic particles, significantly higher expression levels of *cytb, mtMutS*, *hsp60, hsp70* and *MnSOD* genes (Supplementary Table 4) were also found. On the basis of such results and the determination of the gene expression fold change compared to the control, we report here that *cytb*, *mtMutS*, *hsp70* and *EF1* genes were significantly upregulated in the corals exposed to medium concentrations of microplastic particles after 10 days of experiment (Fig. 5a). At the same time, when compared to the control, the genes *cytb, mtMutS*, *hsp60, hsp70* and *MnSOD* were also upregulated in corals exposed to the highest microplastic concentration, especially for *hsp70* and *MnSOD* genes (3.3 and 12.2-fold changes, respectively, Fig. 5a).

The analysis of oxidative DNA damage in coral tissues measured through the concentrations of 8-hydroxydeoxyguanosine (8-OHdG) revealed a significant effect of microplastics exposure (F_3,15_ = 9.22, *P* = 0.0012). Corals exposed to medium and high concentrations of microplastic particles, were characterized by 8-OHdG levels significantly higher than in the control (*P* = 0.0076 and *P* = 0.0021, respectively). Conversely, the 8-OHdG levels in corals exposed to low microplastic concentrations were not significantly different respect to the control (*P* = 0.39; Fig. 5b).

#### Microbial abundance in seawater and the *C. rubrum* microbiome

Prokaryotic abundance in seawater containing microplastics varied significantly among treatments (F_4,20_ = 16.64, *P* = 0.001, and increased significantly between the initial and final measurements (F_1,20_ = 102.65, *P* = 0.001). There was no interaction between treatment and time (F_4,20_ = 2.01, *P* = 0.13). All of the Bonferroni-corrected Tukey pairwise tests contrasting microplastic treatments with controls were statistically significant, whereas contrasts among treatments that contained microplastics were not (Supplementary Fig. 3b). The analysis of the coral microbiome revealed that, at the beginning of the experiment (Control t0), all coral branches showed a prokaryotic richness of 38 ± 0.0 Amplicon Sequence Variants (ASVs). After 10 days, prokaryotic ASV richness increased in the control t10 approximately by a factor 2 (61 ± 0.0). In the coral branches exposed to low, medium and high concentrations of microplastic particles, prokaryotic ASV richness further increased (105.5 ± 26.2, 88.5 ± 9.2 and 116.5 ± 14.1, respectively) compared to the controls at time 0 and after 10 days_._

The composition of microbiomes, in terms of ASVs, changed significantly among the corals investigated (PERMANOVA, pseudo-F_4,9_ = 34.97, *P* < 0.001).

**Fig. 6:**
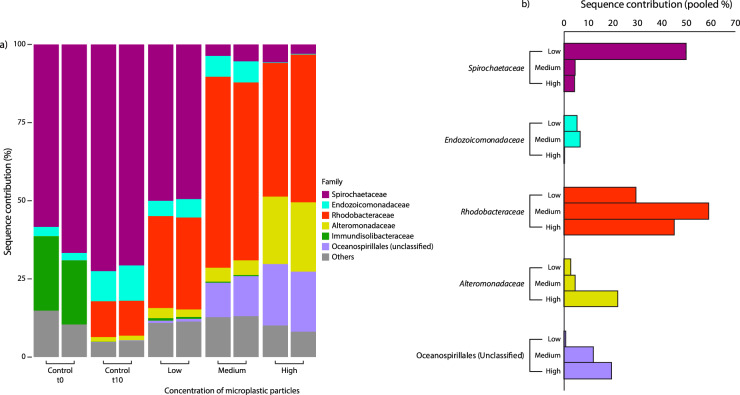
Coral microbiomes at the beginning (Control t0) and after 10 days of experiment (Control t10), and in corals exposed to different concentrations of microplastic particles. **a** Taxonomic composition of microbial assemblages; **b** pooled sequence contribution of relevant families to the taxonomic composition: ubiquitous *Spirochaetaceae* associated with the healthy Mediterranean red corals^57^; *Alteromonadaceae*, *Endozoicomonadaceae*, *Rhodobacteraceae* and *Oceanospirillaceae* typically associated with stressed and diseased corals^57,63^.

The compositions of microbial assemblages in the unexposed corals at the start and after 10 days of experiment were both dominated by *Spirochaetaceae* (mean contribution: 63% and 72%, respectively), but the 10-day controls exhibited increases in *Endozoicomonadaceae* and *Rhodobacteraceae* and the loss of *Immundisolibacteraceae* (Fig. 6a). The microbial assemblages associated with corals exposed to microplastics after 10 days of experiment changed significantly compared to those found in the control t10 (PERMANOVA, pseudo-F_3,7_ = 7.37, *P* = 0.011), but the contribution of *Spirochaetaceae* decreased with increasing microplastic concentrations (50, 5 and 4, at low, medium and high concentrations of microplastic particles, Fig. 6a, b).

Although *Rhodobacteraceae* increased in the controls at the 10th day of the experiment (mean contribution: 11%) compared to control t0, the relative contribution of this family was much higher in the corals exposed to microplastics (ranging from 29 to 59%, respectively, in the treatments with low and medium concentrations of microplastic particles; Fig. 6b).

Besides *Rhodobacteraceae*, the differences in the composition of the coral microbiome at higher concentrations of microplastics were also related to the increase in both unclassified *Oceanospirillaceae* and *Alteromonadaceae* (up to ca. 20% of the assemblage in the treatment with high concentrations of microplastic particles, Fig. 6b).

Two ASVs were present in all samples (“core” ASVs), with similarities >99% with the sequences of the microbiome of Mediterranean gorgonians (belonging to the *Spirochaetaceae* and *Endozoicomonadaceae* families^40^); together, these two core ASVs dominated the assemblages of the control samples (from ca. 56 to 80%), but they decreased when the concentration of microplastics increased (ca. 4% in samples with the highest microplastic particle concentration). After 10 days of incubation, the quantitative relevance of bacteria belonging to the *Endozoicomonadaceae* family decreased from 11% in the control to 0.2% in aquaria containing the highest microplastic particle concentration. A third ASV assigned to the *Rhodobacteraceae* was present in all treatments with microplastics (up to 43% in samples treated with medium concentration of microplastic particles) and was found to be 100% similar to one the clone *LC330582.1 (Uncultured bacterium X151A_June8-1_B06)*^41^.

#### Coral mortality

After 14 days of the experiment, 12 coral branches (6 exposed to medium concentration of microplastic particles and 6 exposed to the high concentration of microplastic particles), no longer showed signs of vitality. These data were also supported by the SEM analyses (Fig. 4), which showed a tissue loss of up to >50%.

## Discussion

The quantification of microplastic concentrations in the Mediterranean Sea and world oceans is a challenging task. Data available in the literature are still largely inconsistent due to the discrepancies in the methodological and sampling protocols used, the different particle sizes investigated and units of the measure adopted. The lowest microplastic concentrations in our experimental systems fall within the range estimated for the area where corals were collected and in other marine environments^7,8,10^. The highest microplastic concentrations used in our experiment, might reflect the contaminations of highly microplastic contaminated habitats and those predicted for open oceans in future scenarios (assuming that present-day concentrations will increase by up to four times current levels by 2030–2060)^9^. Under these conditions, we found that microplastic particles had multiple effects on *Corallium rubrum* and that the extent of this impact increased progressively with increasing particle concentration, determining mortality at the highest contamination levels.

Such effects were not induced by changes in water quality conditions (e.g., temperature, salinity, oxygen concentrations and pH) in the experimental systems since corals unexposed to microplastics were not affected.

We report here that microplastics influenced coral feeding rates. A stimulatory effect potentially driven by the presence of fake food items (i.e., plastic polymers) initially increased feeding rates. Despite corals’ reliance on chemoreception to capture prey^42^, they can exchange microplastics for natural prey because these polymers can contain phagostimulants involved in chemosensory control during feeding^36^. While corals unexposed to microplastics continued to feed without any sign of stress, the rate of ingestion in corals exposed to microplastics decreased significantly over time and with increasing microplastic concentration. After 10 days of exposure to the highest concentrations of microplastics, coral activity decreased to an almost complete cessation of feeding. Feeding impairment may have been due to saturation by the microplastic particles ingested, as previously observed in experiments conducted using scleractinian corals^43^.

Microscopic analyses revealed that microplastics with a wide size range (20–1000 µm) were accumulated by *C. rubrum* over the 2 weeks of the experiment. A fraction of these microplastics, especially of small size (<141 µm; modal size class: 25 µm), was ingested indirectly by the coral through predation on their prey, as revealed by the presence of microplastics of this size in the digestive tract of *A. salina*, thus indicating trophic transfer of microplastic particles^44^.

Previous studies have reported the ingestion of polystyrene by marine copepods^24,45^. Here, we provide evidence, for the first time, that this polymer was ingested preferentially over other types of plastic, although its abundance in the system was relatively low (<10% of the microplastics added to the experimental systems as observed for the marine environment)^8^. This finding is important because we provided a mixture of microplastic particles that mimics the composition currently observed in the oceans^46^. Since the density of polystyrene is similar to that of the larvae considered here and marine zooplankton^47,48^, we argue that its preferential ingestion can be driven by its greater accessibility in the water column. The size range of polystyrene particles used in the experiment was indistinguishable from that of the other polymers considered. Therefore, we exclude that the preferential ingestion by *A. salina* is due to an experimental artefact.

The rates of microplastics ingested via consumption of the contaminated prey (4–5 microplastic particles h^−1^ cm^−2^ of coral considering a coral feeding rate of ca. 37.2–97.0 nauplii h^−1^ cm^−2^) are consistent with the highest values reported for scleractinian corals exposed to microplastics (1.2–55 µg cm^−2^ of coral h^−1^ ^35^ vs. 56–73 µg cm^−2^ of coral h^−1^ in our study). In natural conditions, considering that a colony of *C. rubrum* containing 150 polyps at 40 m depth ingests, on average, 137 ± 32 zooplankton organisms per day^49^, we can estimate that in areas subject to high levels of plastic pollution it can ingest ca. 14–27 microplastic particles daily.

These data indicate that the transfer of microplastics to *C. rubrum*, through the ingestion of contaminated prey, resulting in potential biomagnification of the microplastic particles in the corals, can explain not only one of the causes of the collapse of its feeding activity but can also represent a potential risk for other marine assemblages.

Since *C. rubrum* ingested microplastic particles of size ranging from 20 to 1000 µm and those with a size <141 µm were indirectly transferred by feeding on their prey, we deduce that the microplastics of size ranging from 141 to 1000 µm are directly ingested by *C. rubrum*. Although we removed by sieving the smallest fraction of microplastics (i.e., <20 µm) and rinsed the microplastic particles in prefiltered seawater, we cannot completely exclude the preferential ingestion and effects of nanoparticles, which are present in all aquatic systems. Polypropylene was the most abundant polymer accumulated by the red coral, followed by polystyrene (mostly transferred by predation on zooplankton). Therefore, we can conclude that larger fragments of polypropylene are captured directly by coral polyps. We exclude that the reason for the preferential accumulation of polypropylene in *C. rubrum* is associated with a higher particle contact probability, as polypropylene accounted only for ca. 11% of the total number of microplastic particles. Despite a very similar availability of the different size ranges of microplastics in seawater, the sizes of directly ingested microplastics by *C. rubrum*, was consistent with those of their common prey^49^. Therefore, we argue that the reason for the direct ingestion of polypropylene might be linked to the intrinsic chemical and biological characteristics of this polymer (e.g., chemical composition, additives, adsorbed molecules and presence of microbial biofilm) that favours the release of phagostimulants and, consequently, its ingestion^36^.

The bioaccumulation of plastic polymers (including polyethylene, polyethylene terephthalate and polyvinylchloride) by the red coral, may also be partially explained by passive entrapment/catch mechanisms of microplastics transported by currents.

After one week of exposure, microplastics caused direct damage to coral tissues, causing tears and creating incisions down to the skeleton and sclerites (Fig. 4a, b). At the end of the experiment, corals exposed to the highest concentration of microplastics had more than 50% of tissue surface damaged. Previous investigations carried out on tropical corals reported that exposure to microplastics can cause abrasion and tissue necrosis^50^. Regeneration mechanisms can rapidly repair small lesions of coral tissue, but healing time increases proportionately to the extent of lesions^51^. In mass mortality events, tissue necrosis (with large portions of naked coenosarc) have been documented in red corals and other benthic species^52–54^. The mechanical abrasions observed in our study could contribute to the start of necrosis.

The stress generated by microplastic ingestion/accumulation and coenenchyma tear in *C. rubrum* was manifested by the release of mucus a few hours after the start of the exposure to microplastics. Mucus release was observed in stressed scleractinian corals and/or after the exposure to very high concentrations of polyethylene beads^50,55^. Mucus produced by the red coral colonies entrapped microplastic particles and prokaryotic cells, and its density increased with increasing concentrations of microplastics in the system. Mucus production provides a protective barrier against adverse environmental conditions^56^ and represents one of the first signals of coral stress^57^.

Coral stress was also demonstrated at the molecular level, by the oxidative DNA damage and alteration of the expression levels of key genes involved in the folding of new polypeptide chains, antioxidant activity, DNA repair, protein synthesis and electron transport systems. These genes are crucial to guarantee the defence system of coral cells against stress and toxic compounds^58,59^. In particular, an upregulation of the *MnSOD* gene was observed. The enzyme encoded by this gene is involved in the first line of defence against reactive oxygen species (ROS), which accumulate during stress and can denature proteins, thus inducing DNA damage and oxidizing lipidic membranes and proteins within cells^58^. The homeostatic unbalance observed in our study can lead to the accumulation of denatured proteins leading to the upregulation of *hsp70* and *hsp60* genes (especially at the highest microplastic concentrations). These genes have been reported to be involved in the cellular response to stress of marine organisms, including *C. rubrum*^60,61^.

We observed a significant positive correlation between microplastic concentrations and prokaryotic abundance in the seawater surrounding the coral branches. This indicates stimulation of microbial colonization (or contamination) as observed in tropical coral reef ecosystems subjected to plastic waste^38^. The *C. rubrum* tissues surrounding the lesions were densely colonized by microbial cells and it is known that there is a relationship between coenenchyma degradation and alteration of the microbiome, as previously observed in other octocorals (*Eunicea flexuosa* and *Pseudoplexaura porosa)*^57^.

The 16S rRNA metabarcoding analysis carried out in this study revealed a shift in the microbiome composition of corals exposed to microplastics with an increase in bacterial species richness compared to the controls. *Spirochaetaceae*, which is typically associated with healthy red coral communities^40,57^, was the dominant family in the unexposed corals (both at the start and after 10 days of the experiment). The contribution of *Spirochaetaceae* strongly decreased in the corals with increasing microplastic contamination levels, while the *Rhodobacteraceae*, *Alteromanadaceae* and unclassified *Oceanospirillaceae* increased. These taxa are opportunists rather than primary pathogens^62^ as they colonize mucus and compromised tissues^57,63^, and they are thus typically found associated with stressed and diseased corals^64,65^ A single amplicon sequence variant (ASV), largely contributing to the *Rhodobacteraceae* family, was very similar to an OTU found on a prokaryotic biofilm developed in seawater on poly(3-Hydroxybutyrate-co-3-Hydroxyhexanoate) films and associated with the degradation of this plastic material^41^. At the same time, the *Oceanospirillaceae* family was reported to grow on plastics immersed in seawater^66^, so that microplastics can favour the growth and/or transmission of opportunistic prokaryotes, which take advantage of corals suffering from physiological stress.

In our study, bacteria belonging to the *Endozoicomonadaceae* family, which play a key role in the coral physiology^40,67^, almost disappeared at the highest levels of microplastic contamination as observed in different coral species affected by different anthropogenic impacts^68^.

Based on these findings, we argue that prokaryotic proliferation and microbiome shifts of the red coral are a consequence of coral stress, but also a contributory cause of the reduction in coral health associated with the progressive tissue laceration and transmission of opportunistic pathogens growing on microplastic particles.

Overall, our findings provide evidence, for the first time, that microplastics (mostly polypropylene and polystyrene), especially at the highest concentrations tested here, cause multiple negative effects on *C. rubrum*. The responses observed here could also be associated with the presence of chemical additives in the microplastics, which have been reported to be toxic for marine organisms^69,70^).

**Fig. 7:**
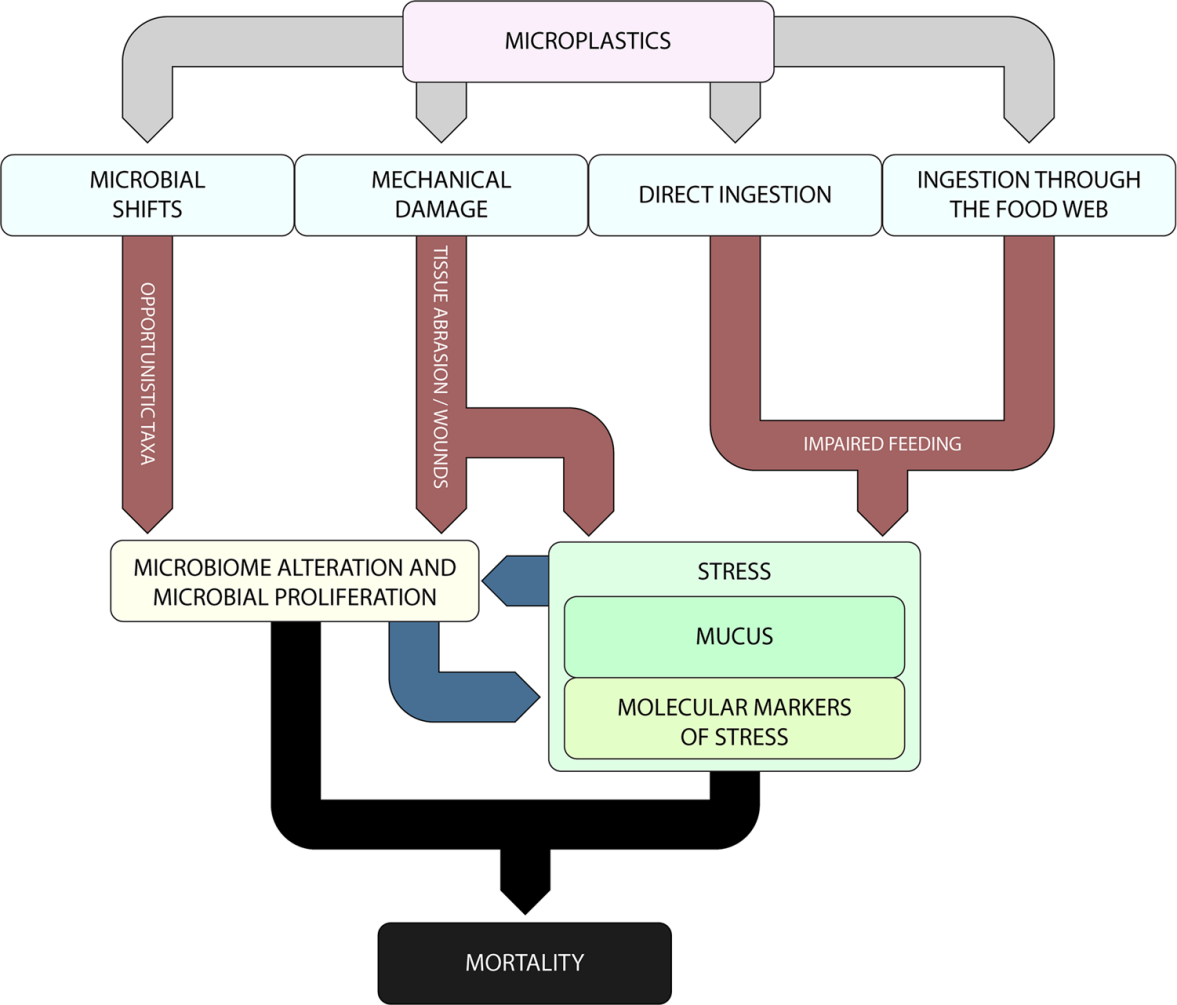
Conceptual model of the multiple biological and physical impacts of microplastics on the red coral. Effects induced by ingestion, physical contact with coral tissue and microbial response to the presence of microplastic particles in the seawater. Reported are the consequent shifts in coral microbiome composition and stress in terms of changes in gene expression and DNA oxidative damage. The combination of all of these processes can ultimately cause coral mortality.

The ingestion and accumulation of microplastics can impair feeding activity (Fig. 7). This effect, combined with the tissue abrasion, caused by the mechanical contact with microplastics, increased the stress levels of *C. rubrum*, which are apparent either macroscopically, by excessive mucus production, and at the molecular level, as shown by the altered cellular response (i.e., decrease of the cell defence against plastic polymers). Further evidence of coral health deterioration caused by microplastic contamination is provided by the proliferation of prokaryotes and, consequently, altered microbiome composition of the corals. Our results reveal an increase of the opportunistic bacterial taxa, which are known to be associated with coral mortality^62,63^.

The biological effects of microplastic pollution identified here (Fig. 7), based on mechanistic evidence of biological responses, are likely to apply to a wide range of marine life forms (e.g., both active and passive filter feeders, sessile predators and zooplankton feeders^7,43,71^). Although the experimental conditions can only simulate natural conditions (where additional factors can interact to determine coral mortality), based on our findings we anticipate that, if microplastic contamination is not stopped, the values predicted for 2030–2060 will be, in many parts of the oceans similar or higher than those used in our experimental systems.

There is also evidence that some marine habitats, such as marine caves, can trap and concentrate marine litter and microplastics, thus increasing the threat for red corals and other vulnerable sessile species inhabiting these systems^53^. In addition, since microplastic concentrations can be high at all depths (due to the lack depth-related patterns^72,73^), we cannot exclude that microplastics may cause biological effects in deep-sea corals similar to those observed in red corals.

We conclude that microplastic contamination has the potential to threaten red corals and other species that share similar characteristics, and that such an impact could act synergistically with climate-driven events (e.g. heat waves), which have been reported to cause mass mortalities in benthic assemblages^52^.

## Methods

### Collection of marine organisms

Marine invertebrates such as *Corallium rubrum* are ideal organisms to perform controlled experiments and to gather useful information on a variety of environmental conditions^74^. This species, whose diet is based on small zooplankton captured with the polyp tentacles, has been already used in long-term experiments^74–76^. Coral specimens were collected in March 2017 at ca. 35-m depth in the Marine Protected Area of Portofino (Punta del Faro, 44°17′41.02 N; 9°13′31.30 E) in the Ligurian Sea (North-Western Mediterranean Sea) by scuba divers (using TRIMIX blending).

### Experimental design

After recovery, the coral specimens were brought to the laboratory and maintained in a tank (30 L) at in situ temperature (13 ± 0.8 °C) and subjected to the continuous flux of natural seawater filtered onto 0.7-µm pore-size membranes (micro-glass fibre paper, Munktell) by using two submersible pumps (Euronatale, 203 V, 50 Hz, 4 Watt).

Sixty coral branches obtained from different colonies, with similar morphology, and a surface of ~2 cm^2^ each, were distributed among 12 experimental tanks, in order to have 5 coral branches per tank (12 L glass tanks, containing on average, 274 ± 26.4 coral polyps each). The corals were acclimatised for 20 days in a temperature-controlled room, and dim light conditions, before starting experiments. Each tank, filled with natural seawater, was equipped with a prefiltered (0.2 µm) channelled aeration system combined with motor-driven paddles in order to create convective currents, which allowed the resuspension of the microplastic mixture, thus ensuring as much as possible a homogeneous distribution of the polymers. This experimental system was designed and set up according to Sutherland et al.^77^. To assess the potential effects of increasing microplastic  microparticles L^−1^ (here defined as low, medium and high concentrations of microplastic particles). We also quantified the exact amount of particles actually interacting with the corals, by discounting the fractions loss due to experimental manipulations (see details in Supplementary Methods). According to the results reported in the Supplementary Results, the systems were responsible for the loss of ca. 40% of the microplastic particles, thus the corals in experimental systems were actually exposed to 60, 300 and 600 microplastic particles per litre (to which we referred the low, medium and high concentrations).

The highest concentrations of microplastics (up to 600 microplastic particles per litre) can reflect future contamination on the basis of estimates obtained by numerical models^9^, whereas the low and medium concentrations have been selected to represent highly-contaminated marine habitats, including the areas where the corals were collected (Ligurian Sea)^78,79^. In particular, for the Ligurian Sea, we estimated an average value of 94 microplastic particles L^−1^, based on the concentrations of microplastic particles (>200 µm) determined by Fossi et al.^78,79^, and the most cautionary correction factor (10^5^) calculated by Brandon et al.^10^ for the unaccounted smaller fraction of microplastics (25–75% of the fragments falling approximately in the 20–100 µm dimensional class with median range: 59–116).

Microplastic mixtures were also prepared considering the concentration and composition of dominant polymers in different coastal marine environments, especially in hot spots of microplastic contamination^5,7,8^.

The microplastic mixture added to the tanks was composed of 76.6% polyethylene, 10.9% polypropylene, 7.3% polystyrene, 3.3% polyvinylchloride and 1.8% polyethylene terephthalate particles. These particles were obtained by milling plastic objects from everyday life (i.e., containers, bottles, cups, pipes) according to Paul-Pont et al.^8^ (Supplementary Table 5). Plastic milling was carried out under a laminar flow hood in chilled sterilized and 0.02 µm prefiltered milliQ water. All the tools used for handling plastics were pre-treated with 1% sodium hypochlorite in water and rinsed 10 times with sterilized and 0.02 µm prefiltered milliQ water, and then dried under laminar flow hood. Details on the preparation of microplastic mixtures are reported in the Supplementary Methods.

The low, medium and high concentrations of microplastics were added in triplicate tanks (*n* = 3 for each concentration). Additional systems containing seawater and coral branches without microplastics (*n* = 3, here defined Controls), and seawater added with microplastics (at the highest concentration) without red corals (*n* = 3, here define CTRL MPs) were used as controls. Overall, the experimental setup comprised 15 tanks.

The experiments for assessing the impact of microplastics on red corals started immediately after the microplastic mixture addition (time 0). During the experiment, seawater temperature (range: 13.10 ± 0.01–13.13 ± 0.05 °C), salinity (range: 38.35 ± 0.18–38.65 ± 0.18) and oxygen levels (7.10 ± 0.08–7.36 ± 0.2 mg L^−1^) were monitored daily in all tanks using a probe (YSI Professional Plus, USA) and corals were fed three times a week with 10^3^ *Artemia salina* nauplii L^−1^.

After ten days the condition of corals that were exposed to microplastics was deteriorating, so we collected one coral branch from each tank for molecular analyses (i.e., associated microbiome, gene expression and DNA damage). After 14 days, the experiment was stopped because the coral branches that were exposed to the medium and high microplastic particle concentrations were completely wrapped in mucus, with a large portion of damaged tissue and without polyp activity, therefore corals were defined dead (overall 12 branches, see ‘Results’ section for details). Coral branches in the controls showed no visible signs of necrosis or other macroscopic stress. The tissue remained intact, and the colour unchanged until the end of the experiment.

### Effects of microplastic ingestion

#### Coral feeding activity

To assess the impact of microplastics on feeding activity of *C. rubrum*, analyses based on the use of *Artemia salina* were performed after 2 and 10 days from the start of the experiment (t0) in replicate systems (*n* = 3 for each treatment, *n* = 3 for the controls) according to standard international protocols^80^. The nauplii of *Artemia salina* were reared in the laboratory, incubating 0.5 g of cysts (Ocean Nutrition) in 1 L of seawater filtered onto 0.2-µm filter in a separatory funnel, 2 days before the analysis of feeding rate. At hatching, nauplii were counted and maintained in vials to obtain the concentration of 1000 nauplii L^−1^. To avoid stress, corals (one branch for each tank) were transferred underwater to beakers along with 1 L seawater of each tank. After addition of live *A. salina* nauplii (1000 nauplii L^−1^) to the 1 L beakers containing the coral branches and to the controls, three aliquots of 10 ml seawater were collected after ~30 s from the start of the experiment (t0) and after 2 and 4 h. The remaining nauplii present in each seawater aliquot were counted under a stereomicroscope at ×3.2 magnification (Zeiss Stami 2000). Mean ingestion rates (nauplii removed h^−1^) were determined by linear regression analysis.

### Accumulation of microplastics by *C. rubrum*

To investigate the accumulation of plastic polymers by *C. rubrum* polyps, the number of microplastic particles ingested by coral polyps was evaluated after 14 days of exposure to microplastic mixture, by dissolving polyps and skeleton of the corals (one for each tank at the concentration of 1000 microplastic particles L^−1^) using an acid/base digestion protocol^36^ with some modifications.

To exclude biases on the estimate of the number of microplastic particles actually accumulated within the polyps, coral branches were accurately rinsed with milliQ water and checked under stereomicroscope (at ×50 magnification) for the potential presence of microplastic particles adherent to the coral tissue. Coral branches were then soaked in 5 ml of 4.5% sodium hypochlorite (NaClO) for 24 h and dissolved in 5 ml of 37% HCl for 30 min. Particulate material was retained on a 0.2-μm filter in a vacuum filtration system, and microplastic particles were counted under a stereomicroscope at ×50 magnification. The chemical composition of the polymers ingested by corals was confirmed by FT-IR analyses (Perkin Elmer, software Packages Spectrum 5.3.1). To evaluate possible damage to plastic polymers due to the use of acid/base solutions, we exposed polypropylene, polyethylene, polystyrene, polyvinylchloride and polyethylene terephthalate at the same volume and concentration of NaClO and HCl during digestion of the coral.

### Potential transfer of microplastics by zooplankton

While testing the exposure of the red corals to microplastics, we also determined the rate of microplastic ingestion by *A. salina* nauplii used to feed the red corals, in order to assess their role as potential vectors of microplastics. To do this, additional tanks (*n* = 3) were added with 0.2 µm prefiltered 12 L natural seawater, 1000 nauplii L^−1^ of *A. salina* and the same microplastic mixture used for the experiment on the red corals (at the highest concentration). Three other tanks were used as controls containing 0.2 µm prefiltered 12 L natural seawater and 1000 nauplii L^−1^ of *A. salina*.

Microplastic ingestion by *A. salina* was determined after 2 and 10 days of experiment following the enzymatic digestion protocol previously developed^81^ with some modifications. Such a procedure degrades biological tissues without affecting shape, colour and composition of plastic fragments. Gut contents of 100 individuals of *A. salina* (*n* = 5) were assessed under a stereomicroscope (Leica MZ125) and light microscope (Zeiss Axiovert 200) and photographed with a Zeiss Axiocam digital camera. Afterwards, nauplii were processed immediately according to the modified enzymatic digestion protocol. Nauplii were dried in an oven for 3 h at 60 °C, transferred to glass jars containing a buffer homogenizing solution (400 mM Tris-HCl pH 8, 60 mM EDTA pH 8, 5 M NaCl, SDS 1%) incubated at 50 °C for 15 min and exposed to Proteinase K (1 mg ml^−1^). Then, samples were dried for 2 h at 50 °C, homogenized and re-incubated at 60 °C for 20 min and sonicated on ice (1–2 min) three times. After digestion, the microplastic-containing suspensions were placed in Utermöhl chambers and the microplastics were examined at the inverted light microscope (Leica DMI3000-Bat ×200 magnification) and counted. Microplastics obtained from nauplii digested after 10 days of incubation were also measured and categorized by colours and shape to evaluate the numbers and the size spectra of microplastics ingested by *A. salina* during the experiment.

### Physical impact on coral coenenchyma

#### Scanning electron microscopy (SEM) analyses

To investigate the physical damage of the microplastic mixture on the coral tissues, samples (one branch from each tank including the control) were collected before the start of the experiment (t0), after 7 days and at the end of the experiment and prepared for SEM analyses according to standard protocols^82^ with some modifications. Coral branches were stored in 0.7 µm prefiltered seawater with 4% buffered formalin. After 24 h, samples were washed with 0.7 µm prefiltered seawater and dehydrated for 3 h in 20% ethanol. After 3 h they were washed in the same way and dehydrated in ethanol 50%. After 3 h, samples were stored in 70% ethanol. Samples were stored at +4 °C. We dehydrated samples using different gradients of ethanol solutions (70–80%, 80–90%, 90–95%, 95–99% in 2 days)^82^. Then, samples were dried using HMDS (Hexamethyldisilazane, Aldrich 440191)^83^. Dried samples were mounted on aluminium stubs using Leit-C glue (conductive carbon cement, Neubauer Chemikalien) and sputter-coated with gold. Samples were examined with a Scanning Electron Microscope (Zeiss SUPRA 40). In addition, the tissue damage percentage was assessed on SEM micrographs at ×200 of magnification by using PhotoQuad v1.4 software^84^. Such a software for advanced image processing of 2D photographic quadrat samples, dedicated to ecological applications, was used for the analysis of three randomly selected areas from the apex to the base of each coral rotating it on three sides (*n* = 9). Additional analyses through random SEM observations (*n* = 20) at 3.00KX to 17.00KX of magnification were carried out to determine prokaryotic cell abundances around lesions of corals (*n* = 3) exposed to high concentrations of microplastic particles. Data were standardised to the coral surface analysed.

#### Mucus release and trapped microplastics and prokaryotic cells

To evaluate the first symptoms of coral stress, a photographic report was conducted daily. The abundance of microplastic particles trapped in coral mucus was estimated using an enzymatic digestion protocol^81^ with some modifications. Mucus produced by corals exposed to higher microplastics concentrations was dried in oven at 60 °C for 12 h. After 12 h, five ml of homogenizing solution was added to the samples and incubated at 50 °C for 15 min. Proteinase K (1 mg mL^−1^) was added to the samples, which subsequently were incubated at 50 °C for 2 h. Then, samples were homogenized and incubated again at 60 °C for 20 min, after that samples were sonicated three times (three 1-min treatments using a Branson Sonifier 2200; 60 W). After digestion, microplastics-containing suspension was filtered on 0.2-μm filters in a vacuum filtration system (Whatman, Nuclepore). Filters were analysed at stereomicroscope at ×50 magnification (Zeiss Stemi 2000).

### Stress signals at the molecular level

#### RNA extraction, cDNA synthesis and gene expression level by qPCR

To assess potential changes in the gene expression pattern of *C. rubrum* due to microplastics, total RNA was extracted from ca. 20 mg of tissue (wet weight) from one coral branch randomly collected from each treatment (*n* = 3) and control (*n* = 3) after 10 days of experiment by using Quick-RNA™ MiniPrep (Zymo Research, Freiburg, Germany) according to the manufacturer’s instructions. Total RNA was also extracted from additional samples of coral branches collected randomly at the beginning of the experiment. Once scraped by surgical disposable scalpels (Braun), coral tissues were placed in new 2 ml sterile tubes and washed three times with phosphate-buffered saline (PBS 1×). Samples were centrifuged at 1800 rpm for 10 min in an Eppendorf® 5810r refrigerated centrifuge using a swing-out rotor at 4 °C and, after removing the supernatant, were homogenized for 5 min with a RNase-free sterile glass stick in RNA lysis buffer. Contaminating DNA was degraded by treating each sample with DNase dissolved in RNase-free water included in the kit. For each sample, 250 ng of total RNA extracted was retrotranscribed with an iScript™ cDNA Synthesis kit (Bio-Rad, Milan, Italy), following the manufacturer’s instructions. The reaction was performed on the Veriti™ 96-Well Thermal Cycler (Applied Biosystem, Monza, Italy). To evaluate the efficiency of cDNA synthesis, a PCR was performed with primers of the reference gene, cytochrome oxidase I (COI, Supplementary Table 6). The reaction was carried out using MyTaq™ HS DNA Polymerase (Bioline, Luckenwalde, Germany) on the Veriti™ 96-Well Thermal Cycler (Applied Biosystem, Monza, Italy). The PCR programme consisted of a denaturation step at 95 °C for 1 min, 35 cycles at 95 °C for 45 s, 60 °C for 45 s, and 72 °C for 45 s and a final extension step at 72 °C for 10 min.

The expression levels of the six genes of *hsp70, hsp60, MnSOD, mtMutS*, *EF1* and *cytb*, involved in a broad range of functional responses, such as stress, detoxification processes, and DNA repair, were followed by real-time qPCR to identify potential stress of corals exposed to microplastics^61^. For the *cytb*, target-specific primer pairs were designed with the Primer 3 software (http://primer3.ut.ee^85^) using nucleotide sequences retrieved from the GenBank database for *C. rubrum* as template (https://www.ncbi.nlm.nih.gov/genbank/; Supplementary Table 6). SensiFAST™ SYBR® & Fluorescein mix (Bioline, Luckenwalde, Germany) were used for measuring the levels of mRNAs on CFX Connect™ Real-Time PCR detection system (Biorad, Milan, Italy). Fluorescence was measured using CFX Manager™ software (Biorad, Milan, Italy). All genes tested by qPCR in this study were amplified with primers purchased from Life Technologies/Thermo Fisher Scientific (Milan, Italy). The fold change in target gene mRNA expression of corals exposed to microplastics compared with the control was calculated using the comparative CT method using the 2^−ΔΔCt^ equation^86^. *COI* was used as reference gene for normalising the gene expression analyses.

#### DNA oxidative damage

For evaluating oxidative DNA damage potentially due to microplastic exposure on *C. rubrum*, the content of 8-hydroxydeoxyguanosine (8-OHdG) was analysed. DNA was extracted from 20 mg (wet weight) of tissue randomly collected from one coral branch for each treatment (*n* = 3) and control (*n* = 3) after 10 days of experiment using DNeasy Blood & Tissue Kits (Qiagen, Valencia, CA) and following the manufacturer’s protocol. Finally, samples were kept at −20 °C before subsequent analyses. Nucleic acids extracted (2 μg) were transferred into new 2-ml tubes and incubated for 5 min at 95 °C, then rapidly chilled on ice. Samples were digested to nucleosides by incubating the denatured DNA in sodium acetate 20 mM, pH 5.2 with 2 μl of nuclease P1 (6 U/μl; Merck KGaA, Darmstadt, Germany) for 2 h at 37 °C. Each sample was then incubated with 5 μl alkaline phosphatase (1 U/μl; Roche, Mannheim, Germany) in Tris-HCl 100 mM, pH 7.5 for 1 h at 37 °C. The reaction mixtures were then centrifuged for 5 min at 6000 × *g* and the supernatants tested for DNA oxidation with an OxiSelect™ Oxidative DNA Damage ELISA Kit (8-OHdG Quantitation; Cell Biolabs, CA, USA). As positive control, *Escherichia coli* genomic DNA (2 μg) was incubated in a final concentration of 50 and 100 mM H_2_O_2_ overnight at 37 °C, and subsequently tested.

### Prokaryotic abundance in coral mucus and surrounding seawater

To highlight possible effects in terms of prokaryotic contamination associated with the exposure of the corals to microplastics, we determined prokaryotic abundances in the mucus released by *C. rubrum* and the surrounding seawater.

Prokaryotic abundances in the coral mucus collected from each tank (except for the control where coral mucus was not released) after 14 days of the experiment, were analysed by epifluorescence microscopy. The extraction of prokaryotic cells from the mucus (ca. 1 mL for each tank) was performed using pyrophosphate (final concentration, 5 mM) and ultrasound treatment (three 1-min treatments using a Branson Sonifier 2200; 60 W)^87^. Then, samples were diluted from 50- to 100-fold with sterile water filtered onto 0.2-μm pore-size filters (Anodisc filters; black-stained polycarbonate). The filters were stained using SYBR Green I (10,000× in anhydrous dimethyl sulfoxide, Molecular Probes-Invitrogen) diluted 1:20 in prefiltered TE buffer (pH 7.5) and incubated in the dark for 20 min; a drop (20 µl) of antifade solution (composed of 50% 6.7 mmol L^−1^ phosphate buffer at pH 7.8 and 50% glycerol with the addition of 0.5% ascorbic acid) was laid both on a glass slide and on the filter mounted on it. Prokaryotic counts were performed under epifluorescence microscopy (magnification, ×1000; Zeiss filter set #09, 488009-9901-000, excitation BP 450–490 nm, beam splitter FT 515, emission LP 520), by examining at least 20 fields per slide and counting at least 400 cells per filter.

For the determination of prokaryote abundance in seawater surrounding corals, three replicates of 10 ml of seawater were collected from each tank. Total prokaryotic abundance was determined according to Danovaro^87^. Samples were filtered onto 0.2-μm pore-size filters (Anodisc black-stained polycarbonate filters, Whatman) into a funnel with vacuum pressure no greater than 20 kPa (or 150 mmHg) to avoid cell damage. When the sample had passed through, filters were stained with 20 µl of SYBR Green I (10,000× in anhydrous dimethyl sulfoxide, Molecular Probes-Invitrogen) diluted 1:20 in prefiltered TE buffer (pH 7.5) and incubated in the dark for 20 min. Then, to remove the excess stain, filters were washed three times using 3 ml of Milli-Q water; a drop (20 µl) of antifading solution (composed of 50% 6.7 mmol L^−1^ phosphate buffer at pH 7.8 and 50% glycerol with the addition of 0.5% ascorbic acid) was laid both on a glass slide and on the filter mounted on it. Prokaryotic counts were carried out as described above.

### Microbiome of corals exposed to microplastics

The coral microbiome was analysed immediately before the start of the experiment (before the addition of microplastics) and after 10 days of the experiment, both in replicated coral branches exposed to microplastics and in unexposed corals (Control t10). For the analysis of the microbiome, ca. 20 mg of tissue (wet weight) from one coral branch randomly collected from two tanks of each treatment and control was scraped from the skeleton by using surgical disposable scalpels (Braun) and DNA extraction was performed using the QIAGEN DNeasy Blood & Tissue Kit. Briefly, samples were digested with proteinase K at 56 °C overnight or until the tissue was completely lysed, then samples were processed following the manufacturer’s protocol. Finally, samples were held at −20 °C before PCR amplification and sequencing. The molecular size of the DNA extracts was analysed by agarose gel electrophoresis (1%) and the amount and purity of DNA were determined by Nanodrop spectrophotometer (ND-1000). For PCR amplification of the 16S V3 region, the Bacteria-specific primer pair 805R/341F was chosen with Illumina-specific adapters and barcodes. Sequencing was performed on an Illumina MiSeq platform by LGC Genomics GmbH (Berlin, Germany).

Raw sequencing paired-end reads were first joined using the *bbmerge* tool from the BBMap suite^88^ in a two-step process: reads that did not merge in a first step were quality-trimmed to remove low-quality bases (Q < 10) prior to re-joining to increase the number of merged sequences. Subsequently, joined sequences were submitted to the QIIME2 pipeline, as described by Corinaldesi et al.^89^ Amplicon sequence variants (ASVs) were identified through the DADA2 strategy^90^. The SILVA database v138^91^ was used as reference database for taxonomic affiliation of sequences; briefly, reference 16S sequences contained in the database were trimmed within QIIME2 to the region amplified by sequencing primers and representative ASVs were analysed using the *classify-consensus-vsearch* approach (consensus over 51% of at most 5 best hits) for taxonomic affiliation. Representative sequences were subsequently aligned using the MAFFT aligner within the QIIME2 suite^92^. The alignment was masked and was utilized for the construction of a midpoint-rooted phylogenetic tree using the FastTree software^92^. The ASV abundance table was randomly subsampled to 20,000 sequences after removal of ASVs represented by <10 sequences (to remove potentially spurious or non-informative sequences) and used, together with the rooted phylogenetic tree, to carry out statistical analyses to compare samples^89^.

### Statistics and reproducibility

Differences in feeding rates, prokaryotic abundances in seawater and polymers ingested by corals were analysed with general linear models in R, using two-sided tests of one-way or two-way (Time, Treatment) experimental designs. Each experimental tank was treated as a replicate. Homogeneity of variance and normality was checked using residual and quantile–quantile plots. Where significant interactions occurred, post hoc Tukey multiple comparisons were carried out with Bonferroni adjustments to *P* values. Prokaryote abundance in mucus samples was taken in a balanced fashion across tanks, but one tank returned lower values across all three treatments. For this analysis, among-treatment effects were tested using a mixed model using lme4 in R^93^, treating “tank” as a random effect. Among-treatment differences in the extent of physical damage to corals were assessed using logistic regression which is a particular case of a generalized linear model, where the assumed distribution is binomial and uses a logit link function. The effect sizes among treatments were expressed as percentage differences converted from log-scale parameter estimates. Thus, estimates of 95% confidence limits are asymmetric on the arithmetic scale. Microbiome composition in corals was investigated after pre-treating the subsampled ASV table using the Hellinger transformation^89^ for normalizing the contribution of sequences to ASVs (after subsampling the ASV table to 20,000 sequences). The same treatment was applied to the contribution of sequences to prokaryotic families after subsampling to 20,000 sequences. Compositional and structural differences among treatments in the prokaryotic assemblage were tested using permutational multivariate analysis of variance (PERMANOVA)^94^ and pair-wise comparisons. Significant differences in mRNA expression 8-OHdG levels were determined using one-way ANOVA.

### Reporting summary

Further information on research design is available in the Nature Research Reporting Summary linked to this article.

## Supplementary information

Supplementary Information

Reporting summary

## Data Availability

All relevant data are included in the main text and in the Supplementary Information. Raw sequences data and additional datasets are available at the following links: https://figshare.com/s/ad48bdf75eebff0721b2 and https://figshare.com/s/7a5f36ff865278e9feab, respectively.
