# Supplementary material for: Multiple impacts of microplastics can threaten marine habitat-forming species
Source: Commun Biol. 2021 Mar 30;4:431. doi: 10.1038/s42003-021-01961-1 (PMC8010021; doi:10.1038/s42003-021-01961-1)
Supplement: Supplementary file 1 — Supplementary Information [file 42003_2021_1961_MOESM1_ESM.pdf]

Supplementary Information

Multiple impacts of microplastics can threaten marine habitat-forming species

Cinzia Corinaldesi[1*], Sara Canensi[2], Antonio Dell'Anno[2], Michael Tangherlini[3], Iole Di Capua[4], Stefano Varrella[1], Trevor J. Willis[5], Carlo Cerrano[2], Roberto Danovaro[2,4]

[1]Department of Materials, Environmental Sciences and Urban Planning, Polytechnic University of Marche, Via Brecce Bianche, 60131 Ancona, Italy

[2]Department of Life and Environmental Sciences, Polytechnic University of Marche, Via Brecce Bianche, 60131 Ancona, Italy

[3]Department of Research Infrastructures for Marine Biological Resources, Stazione Zoologica Anton Dohrn, Fano Marine Centre, Viale Adriatico 1-N, 61032 Fano, Italy

[4]Stazione Zoologica Anton Dohrn, Villa Comunale, 80121 Naples, Italy

[5]Department of Integrative Marine Ecology, Stazione Zoologica Anton Dohrn, Fano Marine Centre, Viale Adriatico 1-N, 61032 Fano, Italy

[*]Prof. Cinzia Corinaldesi

e-mail: c.corinaldesi@univpm.it

Supplementary Methods

Supplementary Results

Supplementary Figures 1-4

Supplementary Tables 1-5

Supplementary References

Supplementary Methods

Microplastic mixture, size class distribution and quality check

Plastic particles were sieved onto 1000 µm and 20 µm meshes to obtain homogeneous size classes among different polymers, and these were rinsed 3 times with 0.02 µm pre-filtered milliQ sterilized seawater to exclude as much as possible the presence of nanoparticles. We weighted 1 g of each polymer (stock amount) by using an analytical balance (AS, RADWAG, accuracy ± 0.001 g), then we counted under stereomicroscope at 50× magnification (Zeiss Stami 2000), the number of particles contained in each aliquot. The relative quantity of the different plastic polymers added to the 12 L was estimated on the basis of the weight of the particles contained within the stock amount for each experimental treatment (100, 500 and 1000 microplastic particles L^{-1}), and considering the percentage of each polymer reported for the natural environment (Supplementary Table 5). Before addition to the systems, the different polymers were combined and homogenized to obtain microplastic mixtures.

A further analysis on the microplastic mixtures revealed a very similar abundance of particles for the three size classes selected (20-200 µm, 200-500 µm and 500-1000 µm; variation coefficient <10 %).

The FT-IR spectroscopy analyses (Perkin Elmer, software Packages Spectrum 5.3.1) performed on a subsample of the microplastic mixture showed that the chemical characteristics of the polymers remained identical comparing the samples before and after milling.

Before adding microplastics to each tank, we also tested the potential contamination of the polymers by prokaryotic DNA, by qPCR analyses using the TaqMan technology[1] targeting prokaryotic 16S rRNA genes. The results of qPCR analyses revealed that the copy number of 16S rRNA genes associated with the plastic mixture was always below detection limits.

DNA extraction and quantitative PCR analyses for assessing microplastics' contamination by prokaryotic DNA

DNA extraction from microplastics was performed using QIAGEN DNeasy Blood & Tissue Kit with some modifications. In brief, microplastic mixture samples (≈ 25 mg) were digested in proteinase K at 56 °C overnight. Then, samples were subjected to ultrasound treatment (three 1-min treatments using a Branson Sonifier 2200; 60W), after that, they were processed according to the manufacturer's protocol under laminar flow hood.

The prokaryotic 16S rDNA sequences were amplified using the forward primer, 5′-TCCTACGGGAGGCAGCAGT-3′ and reverse (5′-GGACTACCAGGGTATCTAATCCTGTT-3′)[2]. The TaqMan probe was (5′-CGTATTACCGCGGCTGCTGGCAC-3′), which contained a fluorescent reporter dye (6-carboxyfluorescein) covalently attached to the 5′-end and a fluorescent quencher dye (6-carboxytetramethylrhodamine) attached downstream of the reporter dye. The cycling conditions were: 3 minutes at 95°C, followed by 40 cycles of 15 sec 95°C, 1 minute, 60°C. All of the qPCR reactions were performed in a volume of 15 µL with a CFXConnectTM Real-Time PCR detection system (BioRad, Milan, Italy) using iQ Supermix (2×; Bio-Rad) containing 40 mM Tris-HCl, pH 8.4, 100 mM KCl, 0.4 mM each dNTP (dATP, dCTP, dGTP, dTTP), 50 U mL^{-1} hot-start iTaq DNA polymerase and 6 mM $MgCl_2$. In addition, each reaction mix contained 0.1/0.1 µM primers/probes, and 1 µl of template DNA.

To test for the absence of 16S rDNA gene copies, linear calibration curves were performed from a standard solution of *E. coli* (from 0.2 to 200 pg μL^{-1}).

To test for possible inhibition of qPCR, using undiluted aliquots of extracted DNA, in addition to running all sample extracts in serial 10-fold dilutions. All samples, standards, and negative controls were analysed in triplicate qPCR reactions.

The iCycler software analysis programme was used to calculate the Ct values and generate standard curves. The "Ct cut off value" of analysed samples was set at 35 cycles of the PCR above which any measurement response was considered as amplification or fluorescence artefacts and not due to contamination. Since no contamination was detected in the samples investigated, the procedure utilised here allowed us to demonstrate that the microplastic mixture added to the mesocosms was devoid of any external contamination by prokaryotic DNA.

Monitoring microplastic concentrations in the seawater during the time-course experiment

Changes in microplastic concentrations were monitored in the CTRL MPs (i.e., control tanks containing seawater added with 1000 microplastic particles L^{-1} without corals) at day 1, 7 and 14 of the time course experiment. This system allowed us to quantify the potential loss of microplastic particles from the system (e.g., due to the adsorption onto the tank walls) not attributable to the ingestion by corals. To monitor particle concentration in the CTRL MPs, 1 litre of seawater was filtered at each sampling time onto 20 μm pore size filters (Sefar Nitex, Agrinova) and microplastics were counted under stereomicroscope as reported above. The number of microplastic particles lost during the experiment was determined by difference between the concentration of microplastic particles initially added to the treatments (considering 1000 microplastic particles L^{-1}) and the number of microplastic particles counted in the CTRL MPs at each sampling time.

The amount of microplastic mixture removed by water sampling for the different analyses carried out in the experiment was estimated considering the water volumes collected for such analyses and the abundance of microplastics contained therein (assuming a homogeneous distribution of microplastic particles in the tanks).

The number of microplastic particles, which remained at the end of the experiment in the tanks containing corals exposed to 1000 microplastics, was determined after filtering all seawater of the tanks (onto 20 μm pore size filters) and counting microplastic particles under the microscope.

Supplementary results

Microplastic concentrations in sea water during the time-course experiment

The monitoring of microplastic concentrations in the CTRL MPS (i.e., control tanks containing the highest concentrations of microplastics without coral branches; 1000 microplastic particles L^{-1}) revealed a number of microplastic particles ranging from 608 to 633 L^{-1}, after 1, 7 and 14 days respectively (equivalent to an average recovery of 62% of the amount added at the beginning of the experiment). These data were used to correct the microplastic concentrations, to which corals were exposed in the experimental systems.

At the end of the experiment, we recounted all the particles remaining in the control tanks (CTRL MPS), and we confirmed that, on average, ca. 38% of the initial microplastic mixture was potentially adsorbed to wall tanks. The tanks with the coral branches, at the end of the experiment, contained on average ca. 5% of the total microplastic mixture initially added. We also estimated that ca. 1% of the microplastic mixture was removed by water sampling used for all the analyses, and that, based on the results of our study, most of the microplastics added to the systems were taken by corals and Artemia nauplii used to feed them.

Supplementary Figures

a

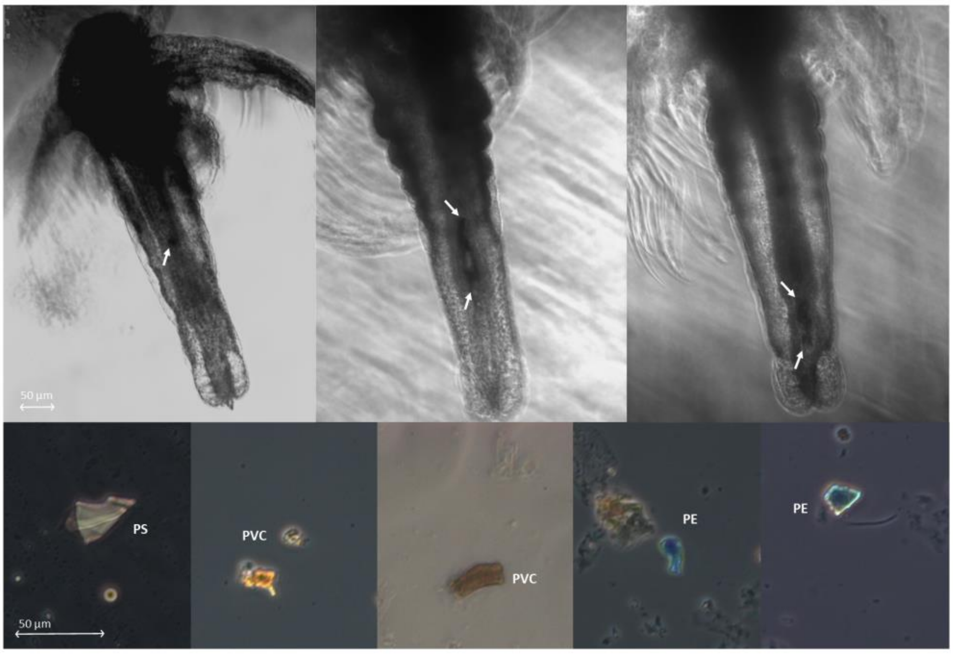

b

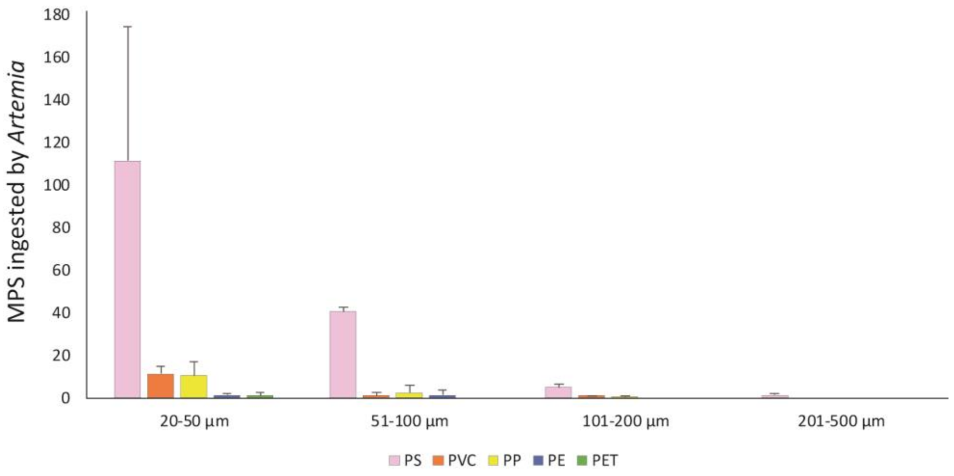

Supplementary Figure 1. Microplastic polymers ingested by *Artemia salina*: (a) images of naupliar stages of *Artemia salina* with microplastic fragments (see the white arrows) in digestive tract; below images of microplastic polymers, after enzymatic digestion, ingested by nauplii during the experiment; **(b)** number of different microplastic polymers ingested by *A. salina* (100 nauplii) according to the different size ranges (20-50 µm, 51-100 µm, 101-200 µm and 201-500 µm) over 10 days of experiment exposed to 1000 microplastic particles L^{-1}. Polymers with size > 200 µm were not ingested by *A. salina*. Plastic abbreviations: PS= polystyrene, PVC= polyvinylchloride,

PE= polyethylene, PP= polypropylene, PET= polyethylene terephthalate**.** Data are represented as mean ± standard deviation.

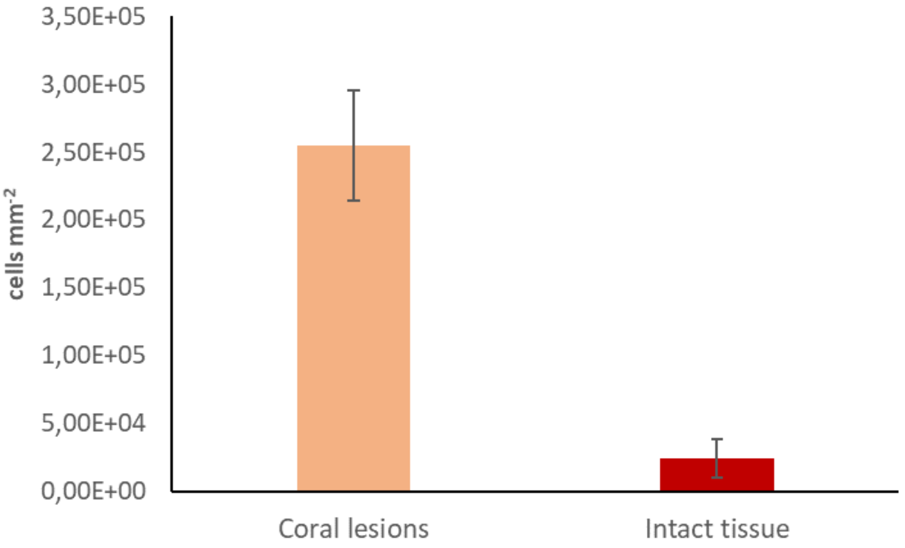

Supplementary Figure 2. Prokaryotic abundance, determined by SEM analyses, around lesions of the corals exposed to high concentration of microplastic particles L^{-1} and on the intact tissue. Data are represented as mean ± standard deviation.

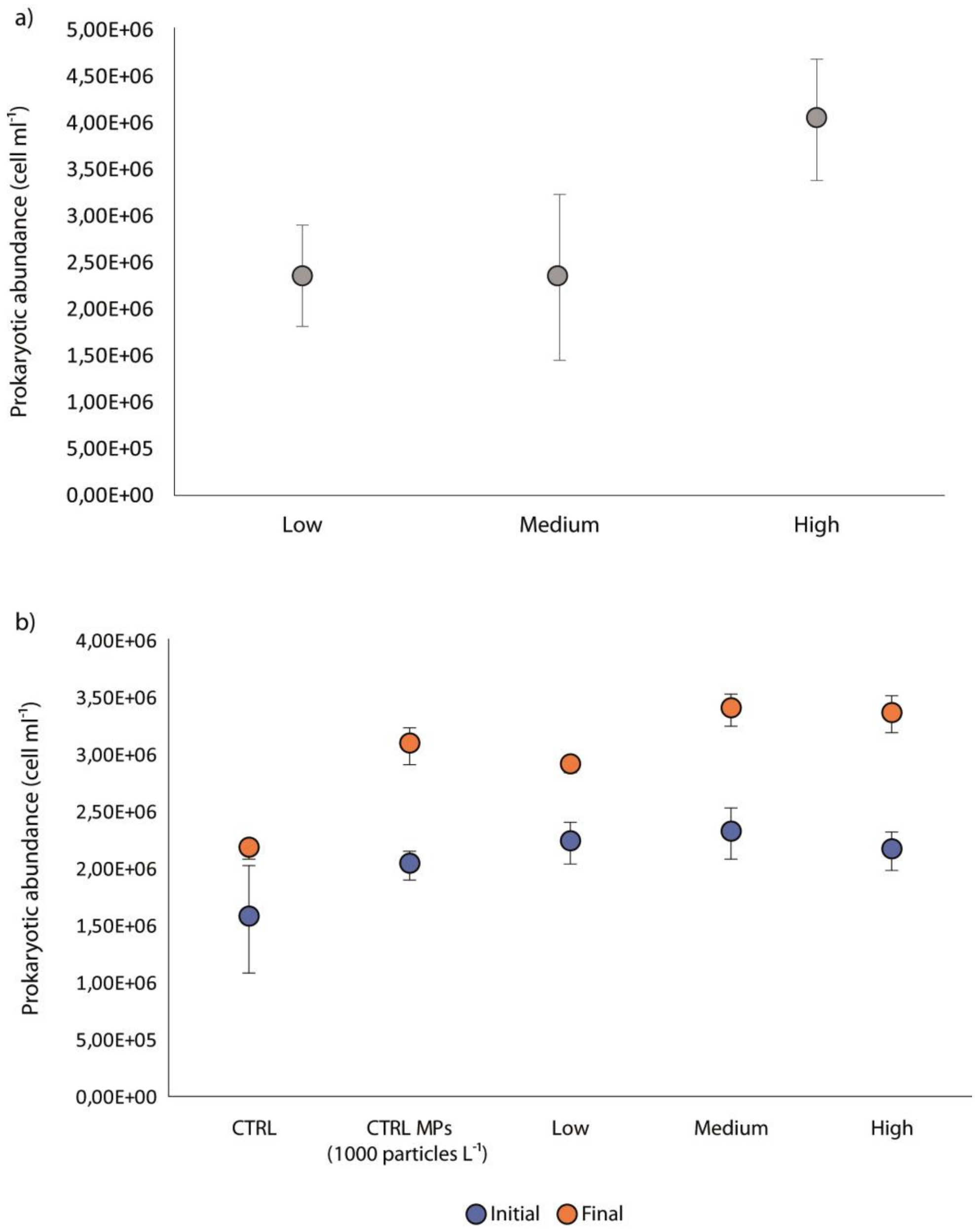

Supplementary Figure 3. Prokaryotic abundance in coral mucus and seawater. a) Total prokaryotic abundances in mucus after 14 days of the experiment at increasing microplastic concentrations (low, medium and high concentrations of microplastic particles). In the controls, coral mucus was not produced. **b)** Total prokaryotic abundance in seawater of the experimental systems with and without microplastics (CTRL, control). The prokaryotic abundance in systems

containing seawater and microplastics (1000 microplastic particles L^{-1}) but without corals (Ctrl MPs) is also shown. Data are represented as mean ± standard deviation.

Supplementary Table 1. Time-course feeding experiments (at t_0 and after 2 and 4 hours) of *C. rubrum* using *A. salina* nauplii in the different microplastic treatments (low, medium and high concentrations of microplastic particles) and in the controls at 2nd and 10th days.

2nd day of experiment		Ctrl	Low concentration	Medium concentration	High concentration
**Replicate**	**Sampling time (h)**	**Nauplii per L of seawater**	**Nauplii per L of seawater**	**Nauplii per L of seawater**	**Nauplii per L of seawater**
r1	0	1033	1125	990	863
r2	0	1116	1387	1084	896
r3	0	950	863	896	829
r1	2	808	567	520	675
r2	2	929	675	691	761
r3	2	688	458	349	589
r1	4	675	292	263	475
r2	4	820	355	398	554
r3	4	530	228	127	396

10th day of experiment	Ctrl	Low concentration	Medium concentration	High concentration

Replicate	Nauplii per L of seawater	Nauplii per L of seawater	Nauplii per L of seawater	Nauplii per L of seawater	nauplii/L of seawater
r1	0	1210	1063	942	1250
r2	0	1286	1228	1039	1330
r3	0	1134	897	844	1170
r1	2	1000	750	858	1200
r2	2	1000	899	955	1250
r3	2	1000	601	761	1150
r1	4	825	525	708	1100
r2	4	850	671	816	1150
r3	4	800	379	601	1050

Supplementary Table 2. Equation of the linear regression, regression coefficient (R), P value of the linear regression model applied to the results of the time-course feeding experiments (see Supplementary Table 1).

**2nd day of experiment**			
**Treatment**	**Linear regression equation**	**R**	**P**
**Ctrl**	y=-89.58x+1018.05	0.82	0.0059
**Low concentration**	y=-208.33x+1077.77	0.91	0.0005
**Medium concentration**	y=-181.87x+954.58	0.92	0.0003
**High concentration**	y=-96.87x+864.58	0.93	0.0001

**10th day of experiment**			
**Treatment**	**Linear regression model**	**R**	**F**
**Ctrl**	y=-96.25x+1204.16	0.97	1.35683E-05
**Low concentration**	y=-134.37x+1047.91	0.86	0.0025
**Medium concentration**	y=-58.33x+952.77	0.75	0.0198
**High concentration**	Y=-37.50x+1258.33	0.76	0.0165

Supplementary Table 3. Results of Student's t-test comparing gene expression levels analysed from the controls (i.e., samples not exposed to microplastic particles) at the beginning (T_0) and after 10 days (T_{10}).

	cytb		*MtMutS*		*hsp70*		*hsp60*		*EF1*		*MnSOD*	
	p-value	***t-value***	***p-value***	***t-value***	***p-value***	***t-value***	***p-value***	***t-value***	***p-value***	***t-value***	***p-value***	***t-value***
**T_0 vs T_{10}**	0.1864	1.5930	0.1555	1.7470	0.1397	1.8390	0.0728	2.420	0.0212	3.6810	0.9529	0.0628

Supplementary Table 4. Results of one-way ANOVA showing the effects of treatment (low, medium and high concentrations of microplastic particles) on the expression levels of *cytb*, *MtMutS*, *hsp70*, *hsp60*, *EF1*, *MnSOD* genes compared to control, and multiple comparisons test between control and different levels of treatment.

					Bonferroni-adjusted multiple comparisons					
**Source**	**df**	**SS**	**F**	**P**	**control vs low conc.**		**control vs medium conc.**		**control vs high conc.**	
cytb					*p-value*	*t-value*	*p-value*	*t-value*	*p-value*	*t-value*
Treatment	3	0.3161	12.78	P=0.0020	>0.9999	0.9199	0.0292	3.374	0.0015	5.609
Residual	8	0.06594								
MtMutS										
Treatment	3	0.0733	6.012	P=0.0190	>0.9999	0.4993	0.0307	3.340	0.0430	3.114
Residual	8	0.0325								
hsp70										
Treatment	3	1.57E-04	10.52	P=0.0038	>0.9999	0.938	0.0163	3.658	0.0026	5.156
Residual	8	3.99E-05								
hsp60										
Treatment	3	2.39E-05	15.62	P=0.0010	>0.2179	1.923	>0.9999	0.0235	0.0050	4.642
Residual	8	4.84E-06								
EF1										
Treatment	3	5.70E-08	378.1	P<0.0001	>0.9999	0.6218	<0.0001	28.04	>0.9769	1.047
Residual	8	4.02E-10								
MnSOD										
Treatment	3	3.55E-07	14.03	P<0.0001	>0.9999	0.3015	>0.9999	0.4338	0.0017	5.530
Residual	8	5.91E-08								

Supplementary Table 5. Plastic polymers added to the systems, density and contribution (%) of each polymer in the mixture.

**Polymers**	**Density (g cm^{-3})**	**%**
1. Polyethylene (PE)	0.91-0.97	76.6
2. Polypropylene (PP)	0.89-0.92	10.9
3. Polystyrene (PS)	1.04–1.1	7.3
4. Polyvinyl chloride (PVC)	1.16–1.58	3.3
5. Polyethylene terephthalate (PET)	1.37–1.45	1.8

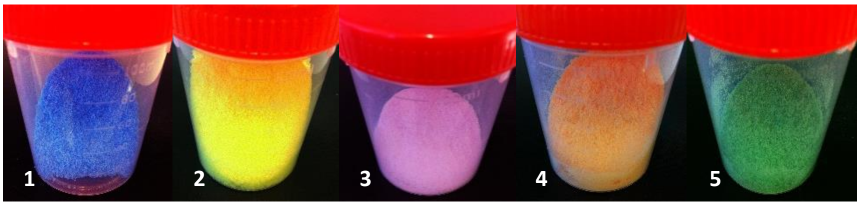

Supplementary Table 6. Gene names, acronyms, functions and primer sequences (5'=>3') are reported for the reference gene and the genes analysed. *primer sequences retrieved from Haguenauer et al. [3]

Gene and acronym	Primer	Sequence (5'=>3')	Function	References
**Cytochrome oxidase I** (COI)*	Cr_COI_For<br>Cr_COI_Rev	GTTCCCACCGGGATTAAGAT<br>CTCCGGTTAAACCACCAATG		
**Heat shock protein 70** (*hsp70*)*	Cr_HSP70_For<br>Cr_HSP70_Rev	TCGACCCAAAGTTGAAGTCC<br>TGCATCTTTTGTCGCTTGAC	chaperonin involved in folding new polipeptide chains	4
**Heat shock protein 60** (*hsp60*)*	Cr_HSP60_For<br>Cr_HSP60_Rev	TATCGCCAAGGAAGGTTTTG<br>TCTTCCGGGGTTGTTACTTG	chaperonin involved in folding new polipeptide chains	4
**Manganese superoxide dismutase** (*MnSOD*)*	Cr_MnSOD_For<br>Cr_MnSOD_Rev	ATGGGGATGGCTTGGTTATT<br>ATACCGAACAAAGGCACCAG	antioxidant activity, scavenger of free radicals	5
**Mismatch repair protein** (*mtMutS*)*	Cr_MtMus_For<br>Cr_MtMus_Rev	ATAAGCCGGATGTTCCTAGTGTA<br>CCATTTGAAGCAAGGATCTTTTA	DNA repair	6
**Elongation factor-1** (*EF1*)*	Cr_EF1_For<br>Cr_EF1_Rev	CTCCATCTGCCATTTCCACT<br>GCTGCTTTTGGTGGATCATT	delivery of aminoacylated tRNA to the elongating ribosomes	7

**Cytochrome b** (*cytb*)	Cr_Cytb_F2<br>Cr_Cytb_R2	TGGGAGCTAGTATCTTGGTGC<br>GGTTCCTCTACCGGGTTAGC	Component of the electron transport system	8

Supplementary References

1. Takai, K., Horikoshi, K. Rapid detection and quantification of members of the archaeal community by quantitative PCR using fluorogenic probes. *App. Env. Microbiol.* **66**, 5066-5072 (2000).

2. Nadkarni, M. A., Martin, F. E., Jacques, N. A., Hunter, N. Determination of bacterial load by real-time PCR using a broad-range (universal) probe and primers set. *Microbiology*, **148**(1), 257-266 (2002).

3. Haguenauer, A., Zuberer, F., Ledoux, J. B., Aurelle, D. Adaptive abilities of the Mediterranean red coral *Corallium rubrum* in a heterogeneous and changing environment: from population to functional genetics. *J. Exp. Mar. Biol. Ecol.* **449**, 349-357 (2013).

4. Hartl, F. U., Bracher, A., Hayer-Hartl, M. Molecular chaperones in protein folding and proteostasis. *Nature,* **475**(7356), 324 (2011).

5. Sinha, K., Das, J., Pal, P. B., Sil, P. C. Oxidative stress: the mitochondria-dependent and mitochondria-independent pathways of apoptosis. *Arch. Toxicol.* **87**(7), 1157-1180 (2013).

6. Bilewitch, J. P., Degnan, S. M. A unique horizontal gene transfer event has provided the octocoral mitochondrial genome with an active mismatch repair gene that has potential for an unusual self-contained function. *BMC Evol. Biol.* **11**(1), 228 (2011).

7. Sasikumar, A. N., Perez, W. B., Kinzy, T. G. The many roles of the eukaryotic elongation factor 1 complex. *Wiley Interdisciplinary Reviews: RNA*, **3**(4), 543-555 (2012).

8. Finkel, T., Holbrook, N. J. Oxidants, oxidative stress and the biology of ageing. *Nature*, **408**(6809), 239 (2000).
