# Supplementary material for: Multiple impacts of microplastics can threaten marine habitat-forming species
Source: Commun Biol. 2021 Mar 30;4:431. doi: 10.1038/s42003-021-01961-1 (PMC8010021; doi:10.1038/s42003-021-01961-1)
Supplement: Supplementary file 2 — Reporting summary [file 42003_2021_1961_MOESM2_ESM.pdf]

nature research

Corresponding author(s): Cinzia Corinaldesi
Last updated by author(s): Feb 17, 2021

Reporting Summary

Nature Research wishes to improve the reproducibility of the work that we publish. This form provides structure for consistency and transparency in reporting. For further information on Nature Research policies, seeAuthors & Referees and theEditorial Policy Checklist .

Statistics

For all statistical analyses, confirm that the following items are present in the figure legend, table legend, main text, or Methods section.

n/a	Confirmed	
☐	☒	The exact sample size (*n*) for each experimental group/condition, given as a discrete number and unit of measurement
☐	☒	A statement on whether measurements were taken from distinct samples or whether the same sample was measured repeatedly
☐	☒	The statistical test(s) used AND whether they are one- or two-sided<br>*Only common tests should be described solely by name; describe more complex techniques in the Methods section.*
☒	☐	A description of all covariates tested
☐	☒	A description of any assumptions or corrections, such as tests of normality and adjustment for multiple comparisons
☐	☒	A full description of the statistical parameters including central tendency (e.g. means) or other basic estimates (e.g. regression coefficient) AND variation (e.g. standard deviation) or associated estimates of uncertainty (e.g. confidence intervals)
☐	☒	For null hypothesis testing, the test statistic (e.g. *F*, *t*, *r*) with confidence intervals, effect sizes, degrees of freedom and *P* value noted<br>*Give P values as exact values whenever suitable.*
☐	☒	For Bayesian analysis, information on the choice of priors and Markov chain Monte Carlo settings
☒	☐	For hierarchical and complex designs, identification of the appropriate level for tests and full reporting of outcomes
☐	☒	Estimates of effect sizes (e.g. Cohen's *d*, Pearson's *r*), indicating how they were calculated

Our web collection on statistics for biologists contains articles on many of the points above.

Software and code

Policy information about availability of computer code

Data collection	Software used in the present research have been largely described in published literature.
Data analysis	This work utilised commonly used procedures in R, including aov and lme4. No custom code was produced.

For manuscripts utilizing custom algorithms or software that are central to the research but not yet described in published literature, software must be made available to editors/reviewers. We strongly encourage code deposition in a community repository (e.g. GitHub). See the Nature Research guidelines for submitting code & software for further information.

Data

Policy information about availability of data

All manuscripts must include a data availability statement. This statement should provide the following information, where applicable:
- Accession codes, unique identifiers, or web links for publicly available datasets
- A list of figures that have associated raw data
- A description of any restrictions on data availability

All relevant data are included in the main text and in the supplementary information. Raw sequences and additionL dataseta are available at the following links: (https://figshare.com/s/ad48bdf75eebff0721b2 and https://figshare.com/s/6dff1da37178435dc5fa, respectively.

Field-specific reporting

Please select the one below that is the best fit for your research. If you are not sure, read the appropriate sections before making your selection.

☐ Life sciences ☐ Behavioural & social sciences ☒ Ecological, evolutionary & environmental sciences

Ecological, evolutionary & environmental sciences study design

All studies must disclose on these points even when the disclosure is negative.

Study description	We investigated the biological effects of microplastics onì the red coral, a long-living, marine habitat-forming species present in different oceans. Here, we demonstrate that corals can preferentially ingest polypropylene and that the ingested microplastics bioaccumulate, causing multiple biological effects, from feeding impairment to mucus production and altered gene expression. Microplastics can also cause tissue abrasions that alter the coral microbiome, with consequent proliferation of opportunistic bacteria, ultimately leading to coral death. Finally, we show that microplastics ingested by zooplankton are transferred to the corals through predation. The multiple biological effects demonstrated here indicate that the current levels of marine microplastic contamination can already threaten many marine life forms, and are expected to alter marine ecosystem functioning in a wide range of habitats by 2030. Experiments were conducted with simple one-factor or two-factor factorial designs. Mixed models were used to determine the possibility of random effects of experimental tanks, even where treatments were balanced among tanks. Sixty coral branches with similar morphology, and a surface of approximately 2 cm^2 each, were distributed among 12 experimental mesocosms (12 L glass tanks), in order to have 5 coral branches per mesocosm (containing on average, 274 ± 26.4 coral polyps each).
Research sample	Here, we tested for the first time the effects of microplastic contamination on the key marine habitat-forming species Corallium rubrum, which is a good model species to gather information on the impact of microplastics in a variety of environmental conditions and across different bathymetric ranges. The genus Corallium is present at almost all latitudes from shallow-water to deep-sea habitats and has a great ecological relevance as it is an ecosystem engineer, thus supporting biodiversity and ecosystem functioning.
Sampling strategy	The experiment was based on 3 replicated mesocosms containing 5 coral branches each per 3 different treatments (3 microplastic mixture concentrations). This sample size was based on the best compromise between statistical relevance ethical issues, given that Corallium rubrum is a endangered IUCN red-listed species. The microplastic mixture was added to the mesocosms in order to mirror the concentrations and composition of dominating polymers reported in marine environment. We used three different concentrations of microplastic mixture. The highest concentrations of microplastics (up to 600 microplastic particles per litre) can reflect future contamination on the basis of estimates obtained by numerical models (Isobe et al. 2019), whereas the low and medium concentrations have been selected to represent highly-contaminated marine habitats, including the areas where the corals were collected (Ligurian Sea, Fossi et al. 2012, 2016). Because coral samples from the same tanks are not independent, we treated tanks as replicates for analyses.
Data collection	We investigated the responses of C. rubrum in terms of feeding activity and defence mechanisms, the tissue damage due to the physical contact with microplastics, responses at the molecular level (i.e., gene expression and DNA damage), and the influence on the coral-associated microbiome. The experimental approach utilized included microscopy-based analyses (including scanning electron microscopy and complementary epifluorescence microscopy techniques). Samples from the time-course experiments were collected by a single operator (Sara Canensi, co-author of the present manuscript) and analysed and recorded by S. Canensi, I. Di Capua and S. Varrella. Metagenomic analyses were performed by LGC Genomics GmbH (Berlin, Germany).Data analyses were performed by C. Corinaldesi, A. Dell'Anno, M. Tangherlini, I. Di Capua, S. Varrella and T. Willis.
Timing and spatial scale	Corals were collected at Portofino in a single sampling event in March 2017.
Data exclusions	No data were excluded.
Reproducibility	All data are contained in the main text, Supplementary Material and Figshare repository.There were no pilot trials, given the vulnerable status of the study species.
Randomization	Coral branches within tanks and treatments were selected randomly for analysis.
Blinding	Blinding was not necessary, but sequence data were obtained without reference to experimental treatments.

Did the study involve field work? ☒ Yes ☐ No

Field work, collection and transport

Field conditions	C. rubrum specimens were sampled in March 2017 at ca. 35 m depth in the Ligurian Sea. Water temperature was 13 ± 0.8 °C.
Location	Coral specimens were collected in the Marine Protected Area of Portofino (Punta del Faro, 44°17’41.02 N; 9°13’31.30 E) in the Ligurian Sea (North Western Mediterranean Sea) by scuba divers (using TRIMIX blending).
Access and import/export	According to the communication we received from the Italian Ministry of the Environment, the restrictive rules for taxa included in Annex D of Presidential Decree 357/97 (and in the EU Directive n. 2010/63) do not apply to C. rubrum and therefore the collection activities and use for scientific experiments do not require authorization.
Disturbance	The disturbance on the C. rubrum colonies of the Portofino Marine Protected Area was minimized by collecting less than 5% of the coral branches from different colonies located in the same area. Being colonial organisms and with high regeneration potential the minimal sampling of coral branches does not affect the health of donor colonies.

Reporting for specific materials, systems and methods

We require information from authors about some types of materials, experimental systems and methods used in many studies. Here, indicate whether each material, system or method listed is relevant to your study. If you are not sure if a list item applies to your research, read the appropriate section before selecting a response.

Materials & experimental systems

n/a	Involved in the study
☒	☐ Antibodies
☒	☐ Eukaryotic cell lines
☒	☐ Palaeontology
☐	☒ Animals and other organisms
☒	☐ Human research participants
☒	☐ Clinical data

Methods

n/a	Involved in the study
☒	☐ ChIP-seq
☒	☐ Flow cytometry
☒	☐ MRI-based neuroimaging

Animals and other organisms

Policy information about studies involving animals; ARRIVE guidelines recommended for reporting animal research

Laboratory animals	This study did not involve laboratory animals.
Wild animals	Corallium rubrum is a sessile invertebrate belonging to the Coralliidae family. Coral specimens were collected by scuba divers (using TRIMIX blending). After recovery, the coral specimens were transported into laboratory in tanks (30 L) for assessing the impact of microplastics.
Field-collected samples	The corals were maintained for 20 days in a temperature-controlled room (13 ± 0.8 °C) in dim light to allow for acclimation before starting the experiment. After 14 days, the experiment was over.
Ethics oversight	No ethical or guidance approval was requireed because C. rubrum is not included in the list of taxa compliant with the restrictive rules for their collection and use according to the italian and EU regulations (see above).

Note that full information on the approval of the study protocol must also be provided in the manuscript.
